# Influence of management practice on the microbiota of a critically endangered species: a longitudinal study of kākāpō chick faeces and associated nest litter

**DOI:** 10.1186/s42523-022-00204-w

**Published:** 2022-09-30

**Authors:** Annie G. West, Andrew Digby, Gavin Lear, Kākāpō Recovery Team, Kākāpō Aspergillosis Research Consortium, Andrew Digby, Doug Armstrong, Darius Armstrong-James, Mike Bromley, Elizabeth Buckley, James Chatterton, Murray P. Cox, Robert A. Cramer, Jodie Crane, Peter K. Dearden, Daryl Eason, Matthew C. Fisher, Sara Gago, Brett Gartrell, Neil J. Gemmell, Travis R. Glare, Joseph Guhlin, Jason Howard, Donnabella Lacap-Bugler, Marissa Le Lec, Xiao Xiao Lin, Lotus Lofgren, John Mackay, Jacques Meis, Kaesi A. Morelli, John Perrott, Megan Petterson, Miguel Quinones-Mateu, Johanna Rhodes, Joanna Roberts, Jason Stajich, Michael W. Taylor, Scott J. Tebbutt, Amber Truter-Meyer, Lydia Uddstrom, Lara Urban, Norman van Rhijn, Deidre Vercoe, Elisa Vesely, Bevan S. Weir, Annie G. West, David J. Winter, Juliana Yeung, Michael W. Taylor

**Affiliations:** 1 School of Biological Sciences, University of Auckland, Private Bag 92019, Auckland, 1142 New Zealand; 2 Department of Conservation, Kākāpō Recovery Team, PO Box 743, Invercargill, New Zealand; 3 School of Natural Sciences, Massey University, Palmerston North, New Zealand; 4 Faculty of Medicine, Imperial College London, London, UK; 5 School of Biological Sciences, The University of Manchester, Manchester, UK; 6 School of Science, Auckland University of Technology, Auckland, New Zealand; 7 Auckland Zoo, Auckland, New Zealand; 8 Geisel School of Medicine, Dartmouth College, Hanover, NH USA; 9 Department of Biochemistry, The University of Otago, Dunedin, New Zealand; 10 Wildbase Research Centre, Massey University, Palmerston North, New Zealand; 11 Department of Anatomy, The University of Otago, Dunedin, New Zealand; 12 Bio-Protection Research Centre, Lincoln University, Lincoln, New Zealand; 13 Novogene, NC USA; 14 Microbiology and Plant Pathology, University of California, Riverside, USA; 15 Dnature Diagnostics and Research, Gisborne, New Zealand; 16 Department of Medical Microbiology and Infectious Diseases Nijmegen, Canisius Wilhelmina Hospital, Nijmegen, The Netherlands; 17 Manaaki Whenua Landcare Research, Lincoln, New Zealand; 18 Department of Microbiology and Immunology, The University of Otago, Dunedin, New Zealand; 19 Flowjoanna, Palmerston North, New Zealand; 20 Department of Medicine, The University of British Columbia, Vancouver, Canada; 21 School of Fundamental Sciences, Massey University, Palmerston North, New Zealand; 22 AgResearch, Mosgiel, New Zealand

**Keywords:** Conservation, Microbiome, Microbiota, Avian, Bird, Threatened, Experimental

## Abstract

**Background:**

The critically endangered kākāpō is a flightless, nocturnal parrot endemic to Aotearoa New Zealand. Recent efforts to describe the gastrointestinal microbial community of this threatened herbivore revealed a low-diversity microbiota that is often dominated by *Escherichia-Shigella* bacteria. Given the importance of associated microbial communities to animal health, and increasing appreciation of their potential relevance to threatened species conservation, we sought to better understand the development of this unusual gut microbiota profile. To this end, we conducted a longitudinal analysis of faecal material collected from kākāpō chicks during the 2019 breeding season, in addition to associated nest litter material.

**Results:**

Using an experimental approach rarely seen in studies of threatened species microbiota, we evaluated the impact of a regular conservation practice on the developing kākāpō microbiota, namely the removal of faecal material from nests. Artificially removing chick faeces from nests had negligible impact on bacterial community diversity for either chicks or nests (*p* > 0.05). However, the gut microbiota did change significantly over time as chick age increased (*p* < 0.01), with an increasing relative abundance of *Escherichia-Shigella coli* over the study period and similar observations for the associated nest litter microbiota (*p* < 0.01). Supplementary feeding substantially altered gut bacterial diversity of kākāpō chicks (*p* < 0.01), characterised by a significant increase in *Lactobacillus* bacteria.

**Conclusions:**

Overall, chick age and hand rearing conditions had the most marked impact on faecal bacterial communities. Similarly, the surrounding nest litter microbiota changed significantly over time since a kākāpō chick was first placed in the nest, though we found no evidence that removal of faecal material influenced the bacterial communities of either litter or faecal samples. Taken together, these observations will inform ongoing conservation and management of this most enigmatic of bird species.

**Supplementary Information:**

The online version contains supplementary material available at 10.1186/s42523-022-00204-w.

## Introduction

The kākāpō (*Strigops habroptilus*) is a critically endangered parrot endemic to Aotearoa New Zealand. Kākāpō are unusual among parrots as they are flightless, nocturnal, exhibit body size sexual dimorphism and undergo lek mating [1]. From only 51 individuals in 1995, today approximately 250 kākāpō are protected on five predator-free islands. These populations are intensively managed by New Zealand’s Department of Conservation (NZDOC, Te Papa Atawhai) to ensure the survival of this unique species.

Kākāpō only mate when plentiful fruit arise following a heavy podocarp mast every 2–4 years, creating highly irregular breeding cycles and slow population growth rates in addition to frequent infertile eggs and embryo deaths [2–4]. In current management practice, fertile eggs are usually removed from the nest shortly before hatching to enhance juvenile survival. Newly hatched chicks are hand reared for one or two days before their release into an appropriate nest, after which chick weight and health are closely monitored. Nest monitoring is a vital component of kākāpō conservation as chick health can decline rapidly due to insufficient feeding, especially if rimu fruit fail to ripen. Large amounts of chick faecal material also accumulate in nests which is regularly removed by NZDOC staff to theoretically prevent disease and infection as only a small number of chicks often survived in previous breeding seasons. However, this practice is not based on scientific testing and it remains unknown whether removing this faecal material (and corresponding microorganisms) may affect development of the kākāpō chick microbiota. Conceivably, removing faecal material may deprive kākāpō chicks of an important environmental source of gut microbiota.

Kākāpō harbour a relatively low-diversity gut microbiota that is frequently dominated (up to ~ 99% of 16S rRNA gene amplicons) by *Escherichia-Shigella* [5–8]. Members of the bacterial phyla *Firmicutes* and *Proteobacteria* often dominate avian intestinal microbiotas, irrespective of host phylogeny or ecology [9–12]. However, an increased abundance of *Enterobacteriaceae* (the family to which *Escherichia-Shigella* belongs) in the gut has been associated with sterility in crested ibis (*Nipponia nippon*) [13] and mortality in juvenile ostrich (*Struthio camelus*) [14]. Nonetheless, *Enterobacteriaceae* frequently inhabit the avian digestive tract [10–12, 15], suggesting a non-pathogenic role for these bacteria in many birds. Marked variation in the relative abundance of *Escherichia-Shigella* among kākāpō individuals has, thus far, not been significantly associated with supplemental feeding, geographic location, age, sex or antibiotic use [6, 7] (West et al. in prep.).

We sought to determine the impact of the current management practice of removing faecal material from nests on the developing kākāpō gut microbiota. In a longitudinal study during the 2019 breeding season, we analysed > 400 samples of chick faeces and corresponding nest litter collected from Whenua Hou/Codfish Island and Pukenui/Anchor Island (Additional file 1: Fig. S1). By experimentally manipulating (removing) faecal matter from some kākāpō nests but not others, we were able to directly evaluate the impact of this practice and inform ongoing conservation efforts.

## Materials and methods

### Sample collection

Faecal and litter material were collected from 67 kākāpō chicks and 34 nests, respectively (Table 1), from Whenua Hou (46° 47′ S, 167° 38′ E) and Pukenui (45° 45′ S, 166° 31′ E) islands (Additional file 1: Fig. S1) between late February–mid May 2019. Samples were placed directly into 5 mL sterile polypropylene tubes containing RNA*later*, then stored overnight at 4 °C and subsequently at − 20 °C until shipping on ice to Waipapa Taumata Rau University of Auckland. We aimed to obtain faecal and litter samples for each chick and nest every fortnight for 10 weeks, with additional samples collected following introduction of a new chick to a nest and follow-up faecal samples collected at least six months later (defined as sub-adult samples). However, the nature of endangered species research is such that sample collection for some chicks and nests occurred less or more frequently than every two weeks, reflecting the workload of island staff and volunteers. An aspergillosis outbreak among kākāpō on Whenua Hou further impacted the study, with all chicks rapidly removed from nests on this island and screened for infection (samples were collected from these Whenua Hou chicks during their stay in the hand rearing facility). Aspergillosis is a respiratory infection caused by *Aspergillus* fungi to which birds are particularly susceptible [16, 17]. Despite these events, we collected and analysed longitudinal samples for more than half of the newly hatched chicks and their corresponding nests.

**Table 1:** Distribution of faecal (chicks) and litter (nest) samples with covariate groupings

	Faecal removal experiment						
	Total	Faeces in	Faeces removed	Mixed	Unknown	Hand rearing	
Faecal samples	287^	64	118	51	8	46	
No. of chicks	67 (35 nests)	15	29	14	2	22 (7*)	
Litter samples	124	44	74	N/A	6	N/A	
No. of nests	34	11	22	N/A	1	N/A	

	Chick age/Days since first chick in nest						
	< 14 days	15–28 days	29–42 days	43–56 days	57–70 days	71–120 days	200 + days
Faecal samples	53	39	54	43	32	21	45
No. of chicks	45	34	46	39	29	16	37
Litter samples	35	32	22	21	8	6	N/A
No. of nests	28	26	19	19	7	4	N/A

	Movement of chicks		Location		Aspergillosis		
	No	Yes	Pukenui	Whenua Hou	Unaffected	Linked	Infected
Faecal samples	92 (14^#^)	181	96	145	188	36	63
No. of chicks	21 (4^#^)	42	24	36	43	7	17
Litter samples	39	85	53	71	80	8	36
No. of nests	12	22	13	21	20	2	12

	Nest type						
	A-frame	Hole	Open	Rock	Tree	Hand rearing	Sub-adult samples
Faecal samples	24	60	4	6	102	46	45
No. of chicks	11	21	2	2	33	23	37
Litter samples	18	37	4	5	60	N/A	N/A
No. of nests	6	9	1	1	17	N/A	N/A

^Of the 287 faecal samples collected, 43 represent faecal material that was pooled where it could not be attributed to a single chick in a nest of multiple juvenile kākāpō

*Indicates number of chicks that were raised entirely in captivity

^#^Faecal samples collected from chicks with no visitors to the nest, but which were placed in a nest previously inhabited by other juveniles (grouped separately for statistical analyses)

Collected metadata included hatch date, sample collection date, location at time of sampling, disease status, nest type (e.g. base of tree, hole within log, behind large rock, under vegetation (‘Open’) or reconstructed by NZDOC staff into an A-Frame) and whether faeces were artificially removed from nests (Additional file 2). Of the 34 nests for which litter samples were successfully analysed, 11 nests were not manipulated (i.e. faeces left in the nest), 22 nests had faecal material removed by NZDOC staff and for one nest this information was not recorded (‘Unknown’; Table 1). Faecal removal varied with chick age and genetic priority but was carried out daily for the first 3 days following hatching or chick introduction to a nest, then every 2–4 days until chicks were 14 days old, every 2–7 days from 15 to 28 days, and every 4–7 days from 29 days until chicks fledged at ~ 10 weeks old. The number of chicks per nest varied, with 10 nests hosting only one chick, 13 nests hosting two and 11 nests hosting three chicks. Metadata were tested as covariates against multiple measures of bacterial diversity and overall microbiota composition.

Of the 67 chicks sampled in this study, 63 were initially hatched and hand reared in on-island captive facilities for 1–2 days during which time they were provided with the commercial feeding formula ‘Kaytee exact Hand Feeding’ (Kaytee, Wisconsin). The remaining four chicks were left to hatch naturally in their respective nests. Seven of the 63 chicks initially hatched in hand rearing facilities were ultimately wholly hand reared until fledging and not placed out in nests on the islands. The remaining 56 chicks initially hatched in hand rearing were then assigned and transferred out to a nest (not necessarily that of their biological mother) with 1–3 chicks occupying a given nest. In response to sub-optimal weight gain or failing health, chicks were often moved among nests or briefly hand reared until a suitable adoptive mother was identified. We refer to the movement of chicks among different nests as ‘chick movement’ (Table 1). Movement among nests was not restricted to the faecal experiment group to which chicks were originally assigned, and hence some chicks were moved among nests that had faeces removed and those that did not (see the ‘Mixed’ category for number of chicks and samples collected under these conditions in Table 1). Movement among nests also meant that the number of chicks for a given nest fluctuated over time and was thus excluded as a testable metadata covariate. Samples collected from chicks being hand reared were not confined to the brief window post-hatching, but occurred throughout the 10-week sampling period as chicks were moved in and out of the facility, particularly during the aspergillosis outbreak.

Between one and six samples were collected per chick or nest, comprising a total of 287 faecal samples and 124 litter samples, respectively (Table 1). Where a faecal sample could not be attributed to a single chick in a nest of multiple individuals, all chicks were listed for that sample, hereafter referred to as a ‘pooled sample’. Faecal samples were collected from one additional nest for which litter samples were not successfully sequenced (Table 1). Overall, 13 nests were located on Pukenui, of which seven nests experienced the movement of chicks and six did not (Table 1). Of the 21 nests located on Whenua Hou, 15 nests experienced chick movement, whilst six nests did not. As many chicks spent some time in and out of the hand-rearing facility, we have faecal samples collected under captive conditions for 22 chicks. Due to the coincident aspergillosis outbreak among kākāpō on Whenua Hou, our data comprise samples from 17 infected chicks and 12 nests where *Aspergillus* was detected in the mother and/or chicks (Table 1). Also included are data from seven chicks and two nests that came into contact with infected individuals but were not diagnosed with aspergillosis or considered a site of infection (‘Linked’, Table 1).

### DNA extraction, PCR and 16S rRNA gene sequencing

DNA was extracted using a modified version of a bead-beating method described by Perry et al. [7], details of which are provided in Additional file 1. This modified protocol reduces the presence of abundant PCR inhibitors and co-precipitation of excess salts.

PCR amplification of the V3-V4 region of the bacterial 16S rRNA gene was performed using primers 341F (5′-TCGTCGGCAGCGTCAGATGTGTATAAGAGACAGCCTACGGGNGGCWGCAG-3′) and 785R (5′-GTCTCGTGGGCTCGGAGATGTGTATAAGAGACAGGGACTACHVGGGTATCTAATCC-3′) [18] with Illumina-compatible Nextera adaptors (underlined). A KAPA 3G Plant PCR kit (Sigma-Aldrich, New Zealand) was used for amplification with thermocycling conditions as described by Perry et al. [7]. Amplicon size and absence of a band for negative controls (DNA extraction controls and no-template PCR controls) were verified on a 1% agarose gel with SYBR Safe DNA Gel Stain (Invitrogen, New Zealand). PCR products were purified using AMPure XP beads (Beckman Coulter, New Zealand) or the Zymo ZR-96 DNA Clean-Up Kit (Ngaio Diagnostics Ltd, New Zealand). DNA concentration was quantified on an EnSpire Multimode Plate Reader (Perkin Elmer) using a Qubit High Sensitivity dsDNA kit (Invitrogen, New Zealand). Samples were normalised to 5 ng/µL for library preparation and sequencing by Auckland Genomics Ltd on an Illumina MiSeq with 2 × 300 bp chemistry.

### Sequence data analysis

16S rRNA gene amplicon sequences were processed in R (version 4.0.1 [19]) using DADA2 (version 1.16 [20]) following the authors’ recommended workflow for paired-end big data [20]. Following initial primer removal, forward and reverse reads were trimmed to 280 bp and 240 bp, respectively. Sequence reads shorter than the truncated value were discarded, as were reads where truncQ < 2 or where the number of expected errors exceeded 3 for forward and reverse reads (-maxEE parameter). Unique amplicon sequence variants (ASVs) were generated, then taxonomy assigned (assignTaxonomy) using the SILVA 138 ribosomal RNA database (silva_nr99_v138_wSpecies_train_set) [21]. Following removal of sequence chimeras and non-target sequences including chloroplasts and mitochondria, the ASV table and taxonomic assignments were merged with corresponding metadata to create separate phyloseq objects for faecal and litter samples using the R (version 4.1.3 [22]) package phyloseq (version 1.38.0 [23]). Low-abundance ASVs (total relative abundance < 0.001%) and ASVs not assigned to phylum level were also discarded. Samples were normalised using scaling with ranked subsampling (SRS), which better preserves the original community structure with fewer subsampling errors than rarefaction [24]. Faecal samples were normalised to 1,200 reads/sample and litter samples to 1,850 reads/sample. ASVs are numbered separately for faecal and litter data in decreasing order of their relative sequence abundance in the respective data set and thus will differ between the two.

To explore variation in bacterial communities among samples grouped by significant covariates (i.e. chick age, faecal removal, location, nest type, disease status), normalised data were transformed to Bray–Curtis and generalised UniFrac (gUniFrac) dissimilarity matrices and ordinated with principal coordinate analysis (PCoA) using the vegan (version 2.5–7 [25]) and GUniFrac (version 1.6 [26]) packages in R. Generalised UniFrac ordinations are included as Additional file 1: Figs. S2 and S3. We tested for significant associations with PERMANOVA (vegan::adonis) where we controlled for variation with repeated sampling (see Additional file 3) as well as among nests (where hand rearing wasn’t a confounding factor). Significant PERMANOVA models were further subjected to pairwise comparison testing using a modified version of the pairwise.adonis2 function in the pairwiseAdonis package (version 0.4 [27]) where we similarly controlled for variation with repeated sampling. We used vegan functions betadisper and permutest to test for homogeneous group dispersion.

Observed ASV richness and Inverse Simpson alpha-diversity indices were calculated on normalised data using phyloseq and associations with metadata covariates tested using Kruskal–Wallis tests (vegan), followed by generalised and standard linear mixed modelling (lme4 package version 1.1–28 [28]), for observed and Inverse Simpson indices respectively, with likelihood ratio testing to assess the significance of mixed models. Post-hoc pairwise comparisons were performed for significant models using Dunn’s test (dunn.test package version 1.3.5 [29]) with Benjamini–Hochberg *p*-value correction [30]. We further tested the effect of chick age and location on the log ratio-transformed relative abundance of abundant bacterial genera (> 3% abundance across dataset) in faecal samples using linear mixed models, controlling for variation among chicks, chick age and location with the lme4 package. Again, we tested for significant relationships using likelihood ratio tests comparing null models with target models for each covariate and genus. A differential abundance analysis was employed using the DESeq2 package (version 1.34.0 [31]) to further investigate ASVs that were differentially abundant in samples collected from chicks under hand rearing versus those out in nests.

Kākāpō chicks were weighed regularly throughout the breeding season, enabling linear comparisons between weight gain (as a proportion of first weight) and gut microbial diversity. We utilised linear modelling for alpha-diversity comparisons (lme4) and PERMANOVA (vegan::adonis2) for analysing associations with beta-diversity.

Finally, we created a separate taxonomic level which concatenated genus- and species-level assignments together and agglomerated the phyloseq object to this taxonomy group. The plyr package (version 1.8.7 [32]) was then employed to group less abundant ASVs into the category ‘Others’ based on a per-species mean relative abundance of < 0.3% for faecal samples and < 0.5% for litter samples. The data were plotted against location for faecal samples and against chick age or ‘days since first chick’ for faecal and litter samples, respectively.

All data were visualised using R packages ggplot2 (version 3.3.5 [33]), ggpubr (version 0.4.0 [34]), cowplot (version 1.1.1 [35]), and Manu (‘kākāpō’ colour palette specifically designed from kākāpō plumage; version 0.0.1 [36, 37]).

## Results

### Bacterial diversity of faecal and litter samples

In total, 33,067,910 raw 16S rRNA gene amplicon paired sequence reads were obtained, with 19,117,949 merged reads remaining after quality and chimera filtering. Of these filtered reads, 99.99% were taxonomically assigned to at least phylum level, spanning 26,602 unique ASVs. Average sequencing depth across all samples was 45,518.93 ± 27,751.97 reads. After removing non-target and low-abundance ASVs, faecal and litter samples were subsampled separately to a minimum read count of 1,200 reads and 1,850 reads, respectively. Four faecal and five litter samples were discarded due to read counts not meeting these thresholds. We ultimately identified 1,065 unique ASVs across 287 faecal samples and 4,403 unique ASVs across 124 litter samples. The number of ASVs per sample ranged from 1 to 180 for faeces and 3–531 for litter, with an average of 37 and 187 ASVs, respectively.

Overall, 96.2% of faecal sample reads could be assigned to genus, but only 78.1% for litter samples. Similarly, 78.7% of faecal sample reads, but only 33.5% for litter, could be assigned to species (however, species-level assignments based solely on 16S rRNA gene data should be interpreted with caution as the inherent conservation of rRNA genes means that fine-scale taxonomic information is not always obtainable from these analyses). *Proteobacteria* was by far the most abundant phylum for both sample types (Table 2). The most prevalent ASVs in faecal samples were F_ASV1_*Escherichia-Shigella coli* (detected in 100% of faecal samples), F_ASV2_*Streptococcus gallolyticus* (58%) and F_ASV4_*Tyzzerella unclassified* (41%). Despite being the third most abundant ASV of the faecal microbiota, F_ASV3_*Lactobacillus gasseri* was only present in 18% of samples (n = 51). L_ASV1_*Escherichia-Shigella coli*, L_ASV2_*Rahnella1 unclassified* and L_ASV7_*Rahnella1 unclassified* were the three most prevalent ASVs across all litter samples (present in 100%, 79% and 77% of samples, respectively).

**Table 2:** Relative abundance of the most abundant bacterial phyla and species across faecal and litter samples

	Most abundant taxa	
Bacterial taxa	Faecal samples (%)	Litter samples (%)
*Proteobacteria*	73.9	75.1
*Enterobacteriaceae*		
*Escherichia-Shigella coli*	57.8	15.9
*Rahnella1* sp*.*	0.65	5.13
*Rhodanobacteraceae*		
*Rhodanobacter* sp*.*	0.35	5.67
*Firmicutes*	23.8	1
*Lachnospiraceae*		
*Tyzzerella* sp*.*	7.9	0.21
*Streptococcaceae*		
*Streptococcus gallolyticus*	5.6	0.61
*Lactobacillaceae*		
*Lactobacillus gasseri*	4.3	0.04
*Bacteroidota*	1	5.68
*Acidobacteriota*	0.89	11.1

Neither bacterial alpha- nor beta-diversity of faecal samples differed significantly with movement of chicks among nests or aspergillosis infection (Table 3). These observations were maintained under generalised (observed richness) and standard (Inverse Simpson diversity) linear mixed modelling (lmm) while additionally accounting for variation with chick age and identity, as well as location and nest residency (1 | location:nest) (Additional file 1: Table S1). Metadata covariates significantly associated with variation in faecal bacterial diversity (alpha and/or beta) included removal of faecal material from nests (‘faecal experiment’), location, chick age, nest type and nest of residence (Table 3; Additional file 1: Table S1). However, the significance of these associations appeared to be driven by samples collected from chicks under hand rearing conditions. Following the exclusion of samples collected in hand rearing, only chick age and nest of residence (Additional file 1: Fig. S4) covariates retained significant associations with bacterial diversity (Table 3), though age had a small effect size (R^2^ = 0.06) compared to nest of residence (R^2^ = 0.36). Both these covariates also exhibited significant heterogeneous group dispersion (Additional file 1: Table S2). Under generalised lmm, chick age was significantly associated with observed species richness while accounting for chick identity and nest residency (Additional file 1: Table S1). However, no significant associations were found between Inverse Simpson diversity and chick age under lmm (Additional file 1: Table S1). We further modelled the overall effect of hand rearing on bacterial alpha-diversity, accounting for sample variation with chick age and identity. Again, hand rearing was significantly associated with observed species richness but not Inverse Simpson diversity (Additional file 1: Table S1). We found no interaction between chick age and hand rearing.

**Table 3:** Statistical outputs for alpha- and beta-diversity measures tested against covariates using Kruskal–Wallis (+ = Wilcoxon) and PERMANOVA analyses, respectively

	Kruskal–Wallis *p*-values for alpha-diversity		PERMANOVA for Bray–Curtis matrices (beta-diversity)		
Covariate	Observed	Inverse Simpson	*p*-value	F	R^2^
*Faecal samples*					
Faecal removal^	0.33/0.003**	0.14/0.21	0.99/ < 0.001***	0.73/10.39	0.01/0.13
Movement	0.46	0.66	0.1	1.98	0.01
Location^	0.15/0.002**	0.02*/0.04*	0.99/ < 0.001***	0.63/22.62	0.002/0.14
Age (continuous factor)^	0.01**/0.007**	0.17/0.11	0.02*/0.06	3.19	0.008
Age (fortnight category)^	< 0.001***/ < 0.001***	0.21/0.09	0.02*/ < 0.001***	2.05/2.78	0.04/0.04
Aspergillosis	0.25	0.32	0.33	1.5	0.008
Nest type^^#^	0.68/0.002**	0.14/0.20	0.02*/ < 0.001***	3.49/8.24	0.01/0.15
Nest of residence^^#^	0.003**/ < 0.001***	< 0.001***/ < 0.001***	< 0.001***/ < 0.001***	2.88/3.38	0.37/0.36
Chick identity	0.19	0.07	1	1.62	0.43
*Litter samples*					
Faecal removal	0.44	0.90	1	1.73	0.02
Movement	0.44	0.12	1	2.26	0.02
Island +	0.79	0.62	1	3.79	0.03
Days since first chick	0.44	0.72	< 0.001***	5.59	0.04
Days (fortnight category)	0.004**	0.12	< 0.001***	2.17	0.08
Aspergillosis	0.52	0.13	1	2.10	0.03
Nest type	0.62	0.85	1	1.75	0.06
Nest	0.52	0.62	1	1.85	0.40

PERMANOVA was performed with 9999 permutations. Significant *p*-values are denoted with asterisks (*p < 0.05, **p < 0.01, ***p < 0.001). *p*-values are Benjamini–Hochberg adjusted. ^ Indicates covariates with two *p*-values for which analyses were performed firstly without hand rearing samples, and then subsequently with their inclusion. # Sub-adult samples were excluded from analyses. Pairwise comparisons for significant tests are provided in Additional file 1: Table S3

**Fig. 1:**
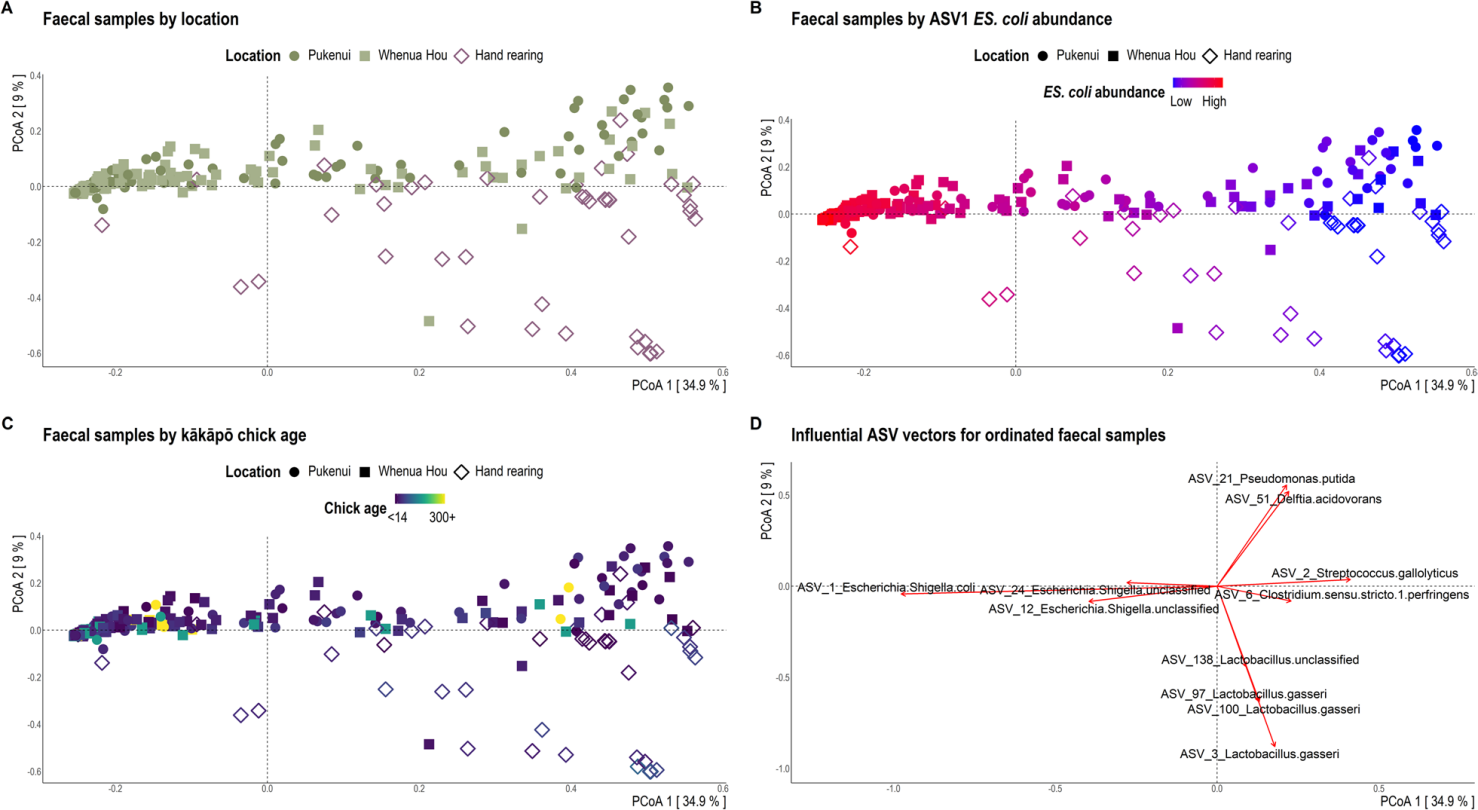
Bray–Curtis dissimilarity distances based on 16S rRNA gene sequences for faecal samples visualised via principal coordinate analysis (PCoA) ordination. Each dot of the PCoA represents the microbiota of a single kākāpō chick faecal sample. Samples are shaped by location and coloured by **A** whether samples were collected while the chick was in the hand rearing facility versus in a nest, **B** relative abundance of *Escherichia-Shigella coli*, and **C** chick age at sample collection. Panel **D** depicts the most influential ASV vectors plotted using the vegan::envfit function

**Fig. 2:**
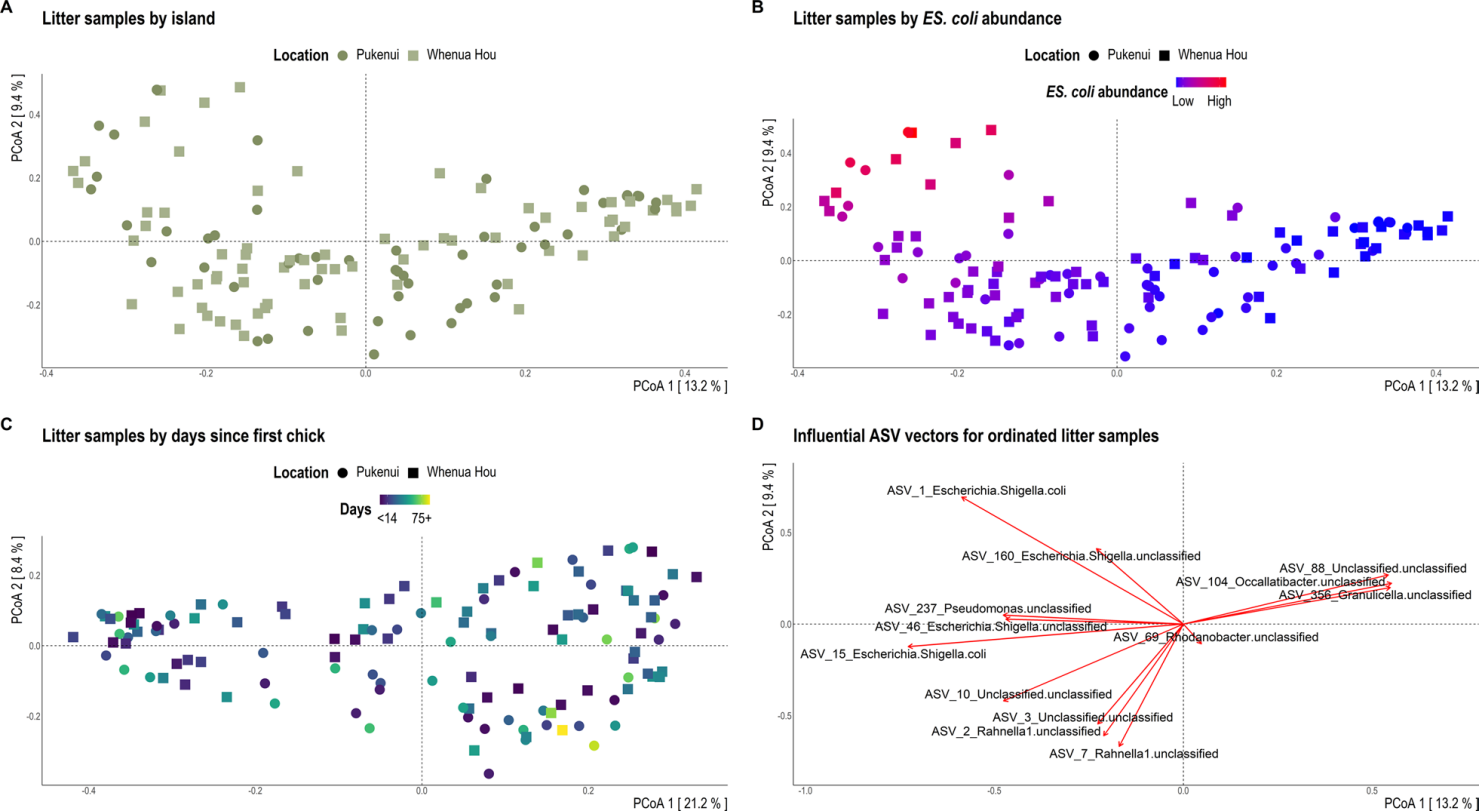
Bray–Curtis dissimilarity distances of 16S rRNA gene sequences for litter samples visualised via principal coordinate analysis (PCoA) ordination. Each dot of the PCoA represents the microbiota of a single nest litter sample. Samples are shaped by island location and coloured by **A** island location, **B** relative abundance of *Escherichia-Shigella* coli, and **C** number of days since the nest sampled first housed a chick. Panel **D** depicts the most influential ASV vectors plotted using the vegan::envfit function

Overall, faecal samples collected from chicks being hand reared were highly dissimilar to all other faecal samples regardless of location (Fig. 1A), nest type (Additional file 1: Fig. S5) or age (Fig. 1C). By contrast, bacterial diversity of litter samples was only significantly associated with temporal collection across all three alpha- and beta-diversity tests (i.e. number of days since a chick was first placed in the nest and subsequent litter samples were collected; Table 3; Additional file 1: Table S1; Fig. 2). Bacterial community separation along PCoA ordination axes was largely due to *Escherichia-Shigella coli* dominance in many kākāpō chick faecal samples (Fig. 1B) and nest litter samples (Fig. 2B).

Chick weight gain over the 10-week sampling period was inversely correlated with both observed species richness (*p* < 0.001, F = 13.97, R^2^ = 0.06, t =  − 3.74) and Inverse Simpson diversity (*p* < 0.001, F = 13.22, R^2^ = 0.06, t =  − 3.64). However, these observations were not maintained for beta-diversity under PERMANOVA (*p* = 0.14, F = 1.58, R^2^ = 0.008).

### Influence of human intervention on the bacterial biota

**Fig. 3:**
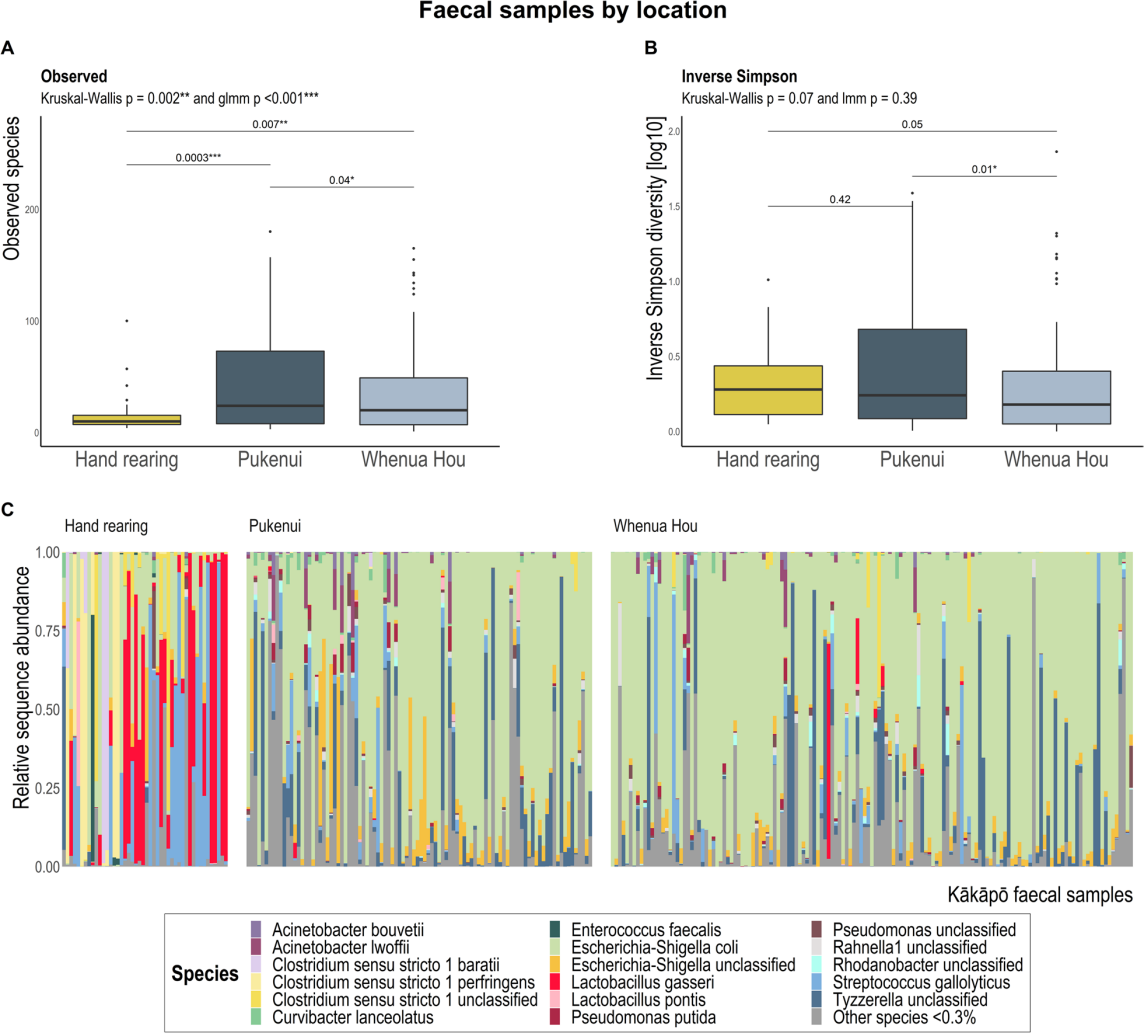
**A** Observed richness and **B** Inverse Simpson alpha-diversity indices for kākāpō chick faecal samples grouped by location. Significant Dunn’s test pairwise comparisons with Benjamini–Hochberg adjustment between location groups in the box-plots are denoted by asterisks (*p < 0.05, **p < 0.01, ***p < 0.001). Boxes represent the median (within-box horizontal line), 25^th^ (lower hinge) and 75^th^ (upper hinge) percentiles. Whiskers extend to the smallest and largest values within 1.5 times interquartile range above the 25^th^ and 75^th^ percentiles, respectively. Data beyond the end of the whiskers are outliers and plotted individually. **C** Species-level taxonomic distribution of bacteria by the relative abundance of 16S rRNA gene sequences within location groups (at time of sampling) for individual kākāpō chick faecal samples (samples for a given chick may include those collected both in captivity and in the nest). Individual samples are ordered within location groups chronologically and alphabetically. Taxa with mean relative 16S rRNA gene sequence abundance < 0.3% are grouped as ‘Other species’

**Fig. 4:**
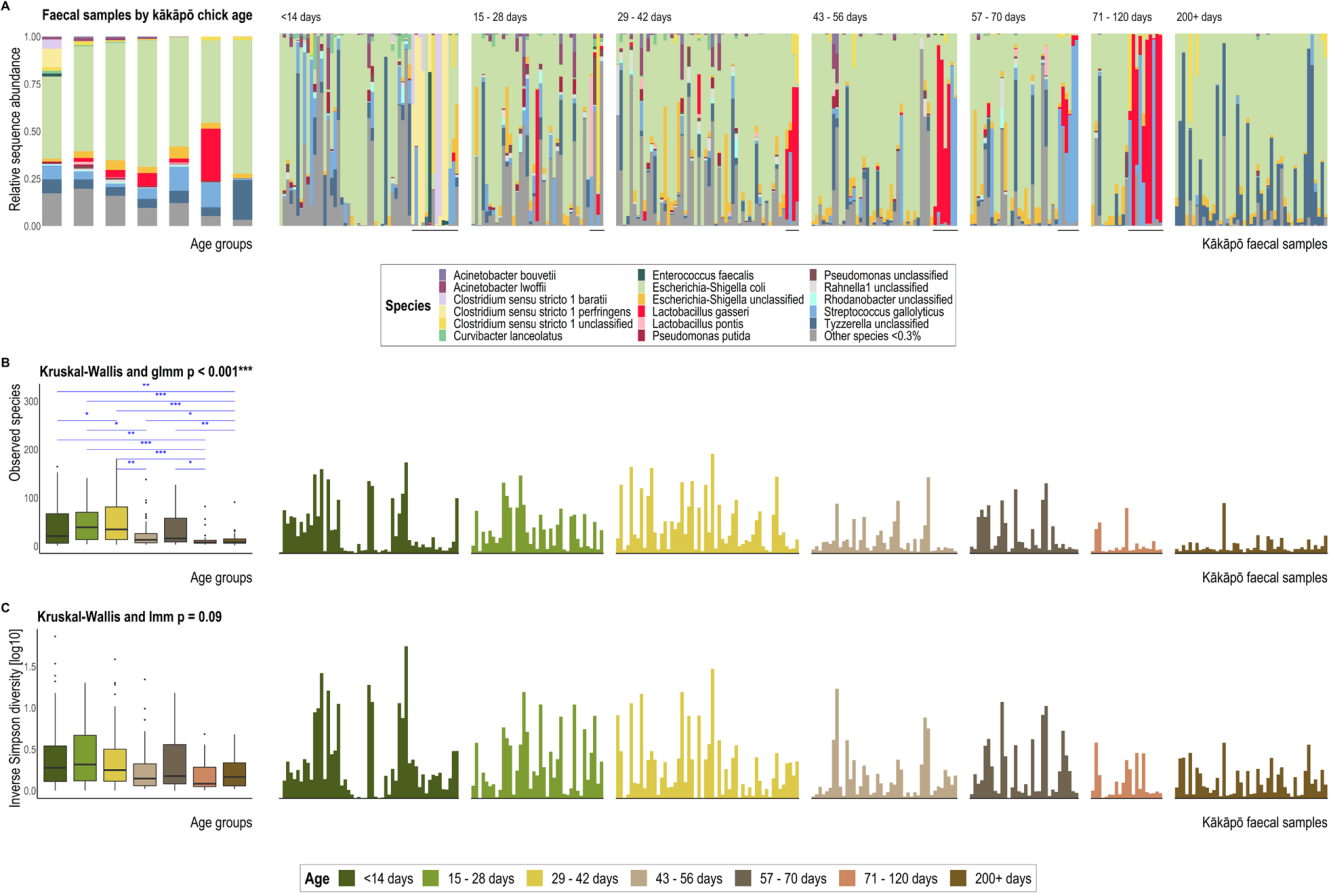
**A** 16S rRNA gene sequence-based taxonomic distribution of bacteria within age groups and individual kākāpō chick faecal samples at the species level. Bars on the aggregated graph (left) are in the same order as the sample-level profile age groups from left to right. Individual samples are ordered within age groups chronologically and alphabetically. Taxa with mean relative 16S rRNA gene sequence abundance < 0.3% are grouped as ‘Other species’. Underlined samples are those collected from chicks being hand reared at the time of collection. **B** Observed richness and **C** Inverse Simpson (plotted on a log10 scale) alpha-diversity indices for kākāpō chick faecal samples grouped by chick age. The y-axes for corresponding bar- and box-plots are identical. Significant Dunn’s test pairwise comparisons with Benjamini–Hochberg adjustment between age groups in the box plot are denoted by asterisks (*p < 0.05, **p < 0.01, ***p < 0.001). Both box- and bar- plots show a decrease in bacterial community diversity with increasing chick age. Box-plot details are as described for Fig. 3

The gut microbiotas of chicks being hand reared in captivity (even for brief periods of time) exhibited significantly lower ASV richness (Fig. 3A), though similar community evenness (Fig. 3B), compared with chicks living in nests, and were dominated by *Clostridium*, *Lactobacillus* and *Streptococcus* bacteria (Figs. 1D, 3C; Additional file 1: Fig. S6). *Lactobacillus gasseri* and *Streptococcus gallolyticus* had significantly greater relative abundance (up to 20-fold based on DESeq differential abundance Wald tests) in faecal samples collected from chicks being hand reared (Fig. 3C; Additional file 1: Fig. S6). One faecal sample dominated by *L. gasseri* but collected from a nest on Whenua Hou represents a 19 d old chick that had been recently moved into the nest from captivity (Fig. 3C). Samples from chicks in the older (43–56, 57–70 and 71–120 d) age categories dominated by *L. gasseri* represent chicks reclaimed from nests and taken back to the hand rearing facility for aspergillosis screening or sub-optimal weight gain (Fig. 4). Comparatively, faecal samples collected from wild chicks had a greater relative abundance of *Escherichia-Shigella*, *Pseudomonas*, *Rhodanobacter, Acidocella, Acinetobacter* and *Tyzzerella* species, among other bacteria (Figs. 1D, 3C; Additional file 1: Fig. S6). The bacterial communities of faecal samples collected from chicks being hand reared had significantly greater dispersion (*p* < 0.001; Additional file 1: Table S2) than the communities of all other faecal samples even when grouped by multiple covariates, including location, faecal experiment and nest type (the only exception where the microbiotas of chicks being hand reared did not have the greatest dispersion was for those grouped by nest of residence). The gut bacterial communities of Pukenui chicks varied slightly more (i.e. had greater dispersion) than chicks on Whenua Hou.

Removing faecal material from nests did not significantly alter the bacterial diversity of either faecal or litter samples (Table 3; Additional file 1: Table S1; Additional file 1: Fig. S7). For this analysis we excluded faecal and litter samples collected from the ‘Unknown’ nest (for which faecal manipulation was not recorded), while faecal samples collected from kākāpō chicks moved among nests under different faecal manipulation regimens were grouped into a ‘Mixed’ category. Samples from chicks under captive, hand rearing conditions had significantly lower bacterial richness than those from chicks located in nests (Additional file 1: Fig. S7A). By contrast, the bacterial richness of ‘Faeces in’, ‘Faeces removed’ and ‘Mixed’ nest groups did not differ significantly from each other (Additional file 1: Tables S1 and S3; Additional file 1: Fig. S7A). Within each faecal experiment group, overall bacterial diversity (measured by Bray–Curtis distances) of faecal and litter samples exhibited a broad range of dissimilarity to other samples within the group (Additional file 1: Fig. S7B). In contrast, gut microbiotas of chicks in captivity were all highly dissimilar to one another, and as a group differed significantly from all other faecal experiment groups (Additional file 1: Table S3; Additional file 1: Fig. S7B).

### Variation over time in the bacterial biota of chicks and nests

There was strong evidence for an age-related shift in faecal bacterial diversity of kākāpō chicks (Table 3; Fig. 4), with a general decline in microbiota richness and diversity with increasing chick age (Fig. 4B, C). In particular, the relative abundance of rarer species (species with mean relative abundance < 0.3% across all faecal samples; Fig. 4A) decreased markedly with a concomitant increase in relative abundances of *Tyzzerella* and *Escherichia-Shigella* species (though this conceivably reflects data compositionality [38]). However, *Escherichia-Shigella* is also relatively abundant in younger age groups and often dominates the kākāpō chick gut microbiota regardless of age (Fig. 4A).

Using linear mixed models (Additional file 1: Table S4), we found a positive relationship between *Escherichia-Shigella* relative abundance and kākāpō chick age (χ^2^ = 30.09, *p* < 0.001). The relative abundance of *Tyzzerella* was also significantly greater in sub-adults than in chicks (χ^2^ = 13.73, *p* = 0.03). Both *Escherichia-Shigella* and *Tyzzerella* were much more abundant in chicks residing in nests than those being hand reared at time of sample collection (χ^2^ = 48.63, *p* < 0.001 and χ^2^ = 42.90, *p* < 0.001, respectively). *Clostridium *sensu stricto* 1* exhibited significantly greater relative abundance in hand rearing samples (χ^2^ = 104.99, *p* < 0.001) and in young kākāpō chicks < 14 d old compared to all subsequent age groups (χ^2^ = 22.06, *p* = 0.001). The former association may be due to most chicks < 14 d old residing in the hand rearing facility. The relative abundance of *Lactobacillus* was also significantly associated with chick age (χ^2^ = 46.65, *p* < 0.001), though *Streptococcus* was not (*p* = 0.19). Both genera were significantly more abundant in chicks being hand reared than those in nests (χ^2^ = 72.64, *p* < 0.001 and χ^2^ = 15.65, *p* < 0.001, respectively). The observation that *Clostridium *sensu stricto* 1*, *Lactobacillus* and *Streptococcus* had significantly greater abundance in samples collected from the captive facility is further supported by differential abundance analyses between hand rearing and wild samples within each age group (Additional file 1: Fig. S6). Samples collected from chicks in nests were significantly enriched with a more diverse assemblage of bacteria than that found in hand rearing samples (Additional file 1: Fig. S6).

**Fig. 5:**
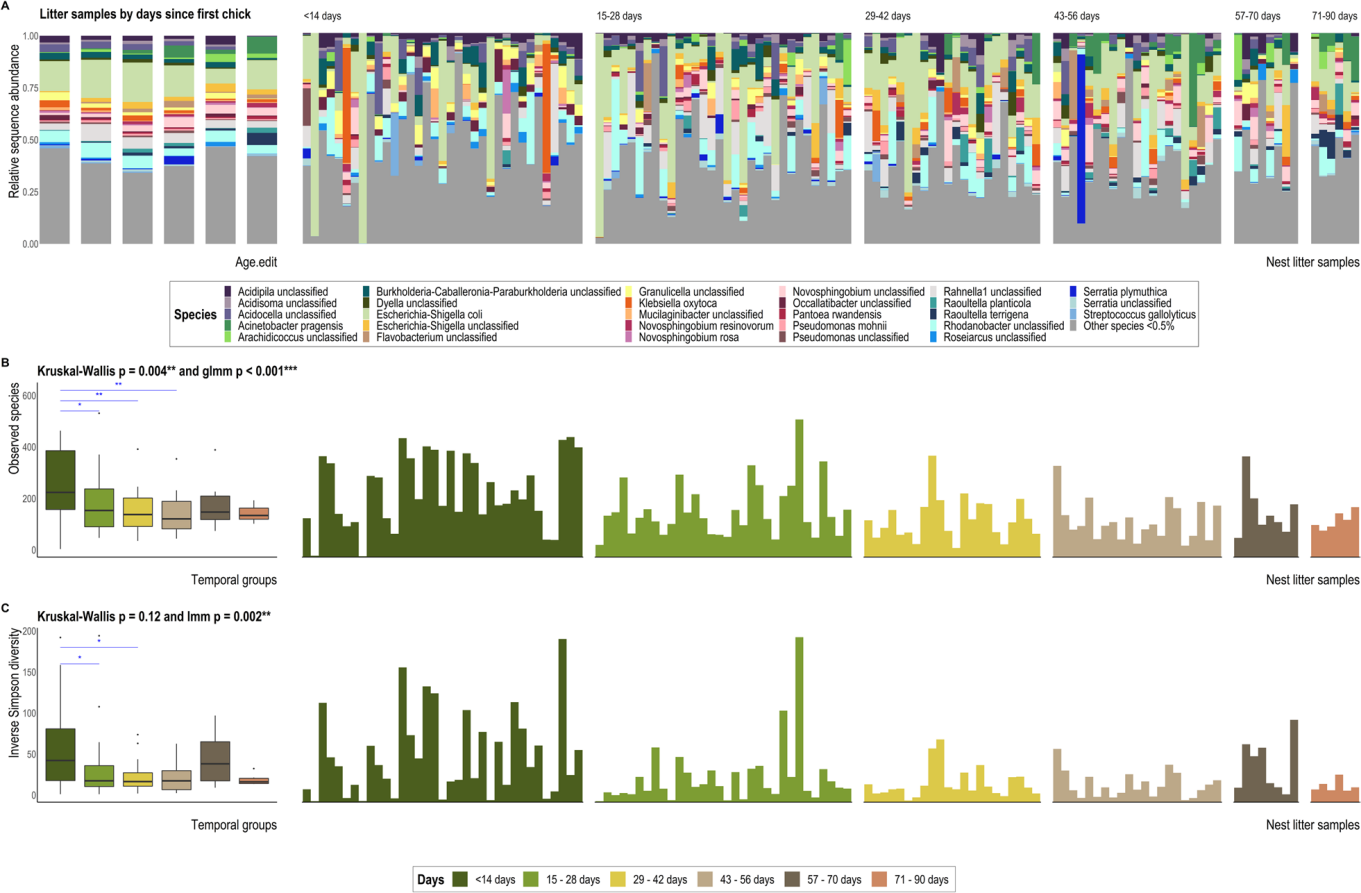
**A** 16S rRNA gene sequence-based taxonomic distribution of bacteria by days since the nest sampled first housed a chick and individual nest litter faecal samples at the species level. Bars on the aggregated graph (left) are in the same order as the sample-level profile groups from left to right. Individual samples are ordered within groups chronologically and alphabetically. Taxa with mean relative 16S rRNA gene sequence abundance < 0.5% are grouped as ‘Other species’. **B** Observed richness and **C** Inverse Simpson (plotted on a log10 scale) alpha-diversity indices for litter samples grouped by days since the first chick was introduced to the nest. The y-axes for corresponding bar- and box-plots are identical. Significant Dunn’s test pairwise comparisons with Benjamini–Hochberg adjustment between groups in the box-plots are denoted by asterisks (*p < 0.05, **p < 0.01, ***p < 0.001). Box-plot details are as described for Fig. 3

Though we observed no significant effect of faecal removal, chick movement, disease (aspergillosis), nest architecture or geographic location on the microbiota of litter samples, we found evidence for a shift in the litter bacterial community over time (since a kākāpō chick was first placed in the nest; Table 3; Additional file 1: Table S1; Figs. 2, 5). The bacterial diversity of litter samples collected in the month following first chick introduction differed significantly from those collected several weeks later, but not to samples collected between 71 and 90 d (Fig. 2C, 5; Additional file 1: Table S3). Bacterial communities of litter samples collected < 14 d following chick introduction had significantly greater dispersion than those of other subsequent time brackets (*p* = 0.0006; Additional file 1: Table S2). Samples collected between 15 and 56 d post chick introduction had significantly lower bacterial diversity than those collected in the first 14 d post-introduction (Fig. 5). Overall, litter samples collected several weeks following chick introduction typically hosted a greater relative abundance of *Yersiniaceae* (bacterial family to which *Rahnella1*, L_ASV3_Unclassified and L_ASV10_Unclassified belong) and *Enterobacteriaceae* compared to those collected < 14 d following introduction of the first chick to the nest (Fig. 2C).

## Discussion

There is mounting evidence that the microbiota of threatened species can be altered by factors including captivity [39–42], habitat fragmentation [43–45], climate change [46, 47] and medical treatment [48, 49], though how such changes may impact animal health remains uncertain [50]. While kākāpō reside on secluded offshore islands with limited human contact, they are, nonetheless, an intensively managed species, particularly during breeding seasons. Kākāpō chicks are monitored closely throughout their first year, with most being hatched in breeding facilities and briefly provided with supplemental feed before being distributed, and later moved, among nests to be cared for by female kākāpō. Nests are also regularly monitored and excess faecal material removed in an attempt to prevent infection in vulnerable chicks. How these combined anthropogenic interventions affect the development of the kākāpō gut microbiota was, until now, largely unknown.

### Hand rearing, but not faecal removal from nests, influences the developing kākāpō gut microbiota

Here we show that the gastrointestinal bacterial community of kākāpō chicks is affected by hand rearing practices but not by removal of faecal material from nests. Removing faecal material from nests also did not affect the composition or diversity of bacterial communities associated with litter samples collected concurrently with chick faecal samples. However, faecal samples collected from chicks under captive conditions at the time differed significantly in bacterial community richness and composition compared to those collected in nests.

Samples associated with hand rearing had lower bacterial richness and were dominated by *Clostridium* sp., *Lactobacillus gasseri* and *Streptococcus gallolyticus*, all members of the *Firmicutes* phylum. We previously showed [6, 7] that several bacterial taxa, identified as *Clostridium*, *Lactobacillus* and *Pseudomonas*, were significantly enriched in the gut microbiota of kākāpō chicks in captivity. However, in neither of the earlier studies did the overall composition of the chick gut microbiota differ significantly from that of adults, nor could it be attributed to supplemental feed. Interestingly, domestic broiler chickens (*Gallus gallus domesticus*) supplied with *Bacillus licheniformis*-fermented products experience a similar increase in *Firmicutes* bacteria, particularly *Lactobacillus* species, and a reduction of *Proteobacteria* members [51, 52]. This fermented product is a listed ingredient of the Kaytee exact Hand Feeding formula fed to kākāpō chicks, as are dried *Bacillus subtilis* and dried *Aspergillus oryzae* fermentation extracts. *L. gasseri* is barely represented in the faecal samples of chicks in nests, suggesting that captive conditions, including supplemental feeding, facilitate its dominance in guts of chicks being hand reared, which is quickly lost upon chicks (re-)entering the nest. Furthermore, birds fed starch-heavy diets, including corn, soy and grain, typically host a significantly greater abundance of *Lactobacillus* than those consuming foliage [12]. The Kaytee feed consumed by young kākāpō contains corn, soy, wheat and oat, thereby facilitating the growth and metabolism of *Lactobacillus* species. We thus find compelling evidence that the Kaytee supplemental feed does, in fact, significantly alter the gut bacterial composition of kākāpō chicks, particularly the abundance of *L. gasseri* (Figs. 1C, 4A). As this study was designed to test the effect of removal faecal material from nests and not rearing method per se, unfortunately only three chicks sampled never spent time in the captive facility during the course of this experiment. Future research specifically regarding the effect of rearing method on the developing kākāpō gut microbiota would benefit from a more appropriately designed experiment for this purpose.

*Clostridium* species were typically identified at greater relative abundance in captive chicks. However, this observation may be confounded by almost all young kākāpō being hatched and reared in captivity before being transferred to wild nests, and may reflect age more than captivity per se. By contrast, *Streptococcus gallolyticus* was reasonably prevalent and abundant across all chick faecal samples, though especially plentiful in samples from hand reared chicks (Figs. 1B, 4A). The distinct presence of *Clostridium* and *Streptococcus* species in young chicks supports the general theory that avian gut *Firmicutes* facilitate chick growth during early development by providing abundant short-chain fatty acids which are easily absorbed across the intestinal epithelium [15, 53–55]. Hird et al. [10] previously identified *Streptococcus* as a core member of the gut microbiota across 59 avian species.

We found an inverse relationship between gut bacterial richness and chick weight gain over the 10-week sampling period. Whether this reflects a biological relationship between weight gain and microbial diversity remains unknown; conceivably, the significance of this association may reflect a decrease in bacterial richness with age rather than a direct relationship with body condition.

Some of the observed variation in gut microbiota diversity among kākāpō chicks is also likely attributable to the mother’s consumption of adult supplementary feed. Female kākāpō are passively provided Harrison’s High Potency Coarse pellets throughout the breeding season at electronic feed stations, and while most mothers preferentially feed their chicks rimu berries, some consumed HPC pellets intermittently. This behaviour varied substantially among females, and even within a single individual throughout the course of this study, and was thus not explicitly tested.

### The kākāpō gut microbiota varies with chick age

Bacterial ASV richness significantly decreased with increasing kākāpō chick age. The bacterial profile of faecal samples collected from sub-adult individuals (at least 200 d post-hatching) resembled those of adult kākāpō, with low bacterial diversity and dominance by *Escherichia-Shigella coli* and/or *Tyzzerella* sp. (West et al., in prep.). Although bacterial richness was significantly decreased in sub-adult faecal samples, community evenness was relatively similar. This is consistent with our observation that only a few taxa often dominated the faecal samples of chicks despite hosting a greater variety of species than their subsequent sub-adult samples. Similar to our observations, the gut microbiotas of recently hatched great tits (*Parus major*), black-legged kittiwakes (*Rissa tridactyla*), dunlin (*Calidris alpina*), red phalaropes (*Phalaropus fulicarius*) and crested ibis (*Nipponia nippon*) host a diverse assemblage of bacteria with considerable inter-individual variation [13, 54, 56]. Moreover, the nest itself can influence the gut microbiota of juvenile great tits, in which microbiotas of foster chicks diverged from that of their true siblings and were more similar within the same nest [54]. In our study, nest of residence significantly influenced bacterial alpha- and beta-diversity in the kākāpō gut. However, we hesitate to interpret these results further given sample sizes among nests varied substantially and exhibited significant heterogeneous dispersion. Given the apparent significance of nest of residence, source tracking analysis may be worth exploring in the future in order to determine the extent of microbial input from the nest environment to the chicks.

The observation that samples from chicks being hand reared exhibited greater within-group dispersion than those collected from chicks in nests is likely confounded by many hand rearing samples representing chicks < 14 d old, a period during which inter-individual variation is substantially higher than that of older chicks in other avians [13, 15, 54, 56]. That a wide variety of bacteria in chicks are not found in adult conspecifics has also been attributed to rapid colonisation of the gut, followed by a subsequent taxonomic shift towards a stable adult microbiota [54, 56, 57]. These studies mostly observed increases in facultative and obligate anaerobes of *Firmicutes* bacteria as the intestinal environment becomes less aerobic from the respiration of initial colonisers, including aerobic members of the *Enterobacteriaceae* family such as *Escherichia-Shigella* [13, 15, 54, 56]. Unlike other avian hosts, the relative abundance of *Escherichia-Shigella* increased with kākāpō chick age and remained the dominant bacterial genus in sub-adult samples collected more than > 200 d post-hatching (Fig. 4A). The dominance of *Escherichia-Shigella* species in the kākāpō chick gut microbiota, and the presence of F_ASV1_*ES. coli* in 100% of faecal samples, is consistent with previous findings for both adults and chicks [5–7] and suggests this pattern represents a typical “wild” bacterial profile for the kākāpō species. It’s possible that, given the long-standing geographic isolation of Aotearoa New Zealand, we find unique ecological systems in the kākāpō gut that are not currently found in other avian species.

### Nest microbiota varies with kākāpō chick occupancy

Similarly to faecal samples, the bacterial richness of nest litter samples decreased over time. The most abundant ASV detected in litter samples, *Escherichia-Shigella coli*, was the same as that identified for faecal samples and was similarly present in 100% of litter samples. We assume that the presence of kākāpō mothers (though not their faeces) and chick faecal material in the nest facilitates the prevalence of this ASV in the nest environment. Litter samples collected after the first month of chick residence generally exhibited an increased relative abundance of *Enterobacteriaceae* and *Yersiniaceae* bacteria (Figs. 2, 5), likely due to accumulation of faecal material over time. The presence of chicks in the nest may have also encouraged proliferation of specific bacteria and thus reduced overall diversity of litter samples with each subsequent collection. Interestingly, the microbiota composition of nest litter did not differ significantly between Pukenui and Whenua Hou islands, perhaps reflecting similar overall vegetation types (dominated by endemic podocarp-hardwood forest [58]). The similarity of litter microbiota from Pukenui and Whenua Hou may also reflect female kākāpō often choosing to nest at the base of hollow rātā trees in the 2019 breeding season. Overall, we find substantial evidence that the presence of kākāpō chicks in nests significantly alters the bacterial community of their surrounding environment.

### Conservation implications

The practice of removing faecal material from nests was discontinued for the 2022 breeding season since it had no discernible impact on overall chick health (as observed by NZDOC staff) nor on the developing kākāpō gut microbiota or that of the surrounding nest environment. However, the marked impact of supplemental feeding on the chick gut microbiota has important implications for management of this critically endangered species and suggests that further consideration of the hand rearing diet for young kākāpō may be warranted. Experimental studies in threatened species research, where feasible, are an important tool to advance our understanding of animal health and aid conservation management programmes [59].

## Concluding remarks

Our primary aim was to explore the influence of current conservation management practices on development of the kākāpō gut microbiota, particularly the regular removal of faecal material from nests during the breeding season. Ultimately, we found no evidence that removing faecal material altered the bacterial composition of faecal or litter samples. However, while previous research regarding the gut microbiota of young kākāpō suggested that neither age nor supplemental feeding significantly influenced bacterial composition [6, 7], the current study provides substantial evidence to revise these earlier findings. Chicks provided supplemental feed in hand rearing facilities hosted significant abundances of *Lactobacillus gasseri* that were absent from wild chicks. This bacterium was likely linked to the Kaytee exact supplemental feed, which contains *Bacillus*-based fermented products. Furthermore, by increasing sample size and longitudinal collection of faecal samples, we revealed that bacterial diversity of the chick gastrointestinal tract decreases significantly with age. This trend is often associated with the gut microbiota of avian nestlings. However, we find a unique shift in taxonomy towards *Proteobacteria* dominance, particularly *Escherichia-Shigella* bacteria, that epitomises the adult kākāpō gut microbiota. Our findings have already provided pertinent information for current conservation efforts of the critically endangered kākāpō and will hopefully aid future research in this field.

## Supplementary Information

**Additional file 1**. Additional information, tables and figures.

**Additional file 2**. Sample metadata table.

**Additional file 3**. RStudio data analysis.

## Data Availability

The raw sequence data are available in the NCBI SRA repository, under Bioproject accession number PRJNA806697. The sample metadata and RStudio data analysis have been included as Additional files 2 and 3, respectively.
