# Supplementary material for: Influence of management practice on the microbiota of a critically endangered species: a longitudinal study of kākāpō chick faeces and associated nest litter
Source: Anim Microbiome. 2022 Sep 30;4:55. doi: 10.1186/s42523-022-00204-w (PMC9523977; doi:10.1186/s42523-022-00204-w)
Supplement: Supplementary file 1 — Additional file 1. Additional information, tables and figures. [file 42523_2022_204_MOESM1_ESM.docx]

Additional file 1: Supplementary Material

*West et al.* Influence of management practice on the microbiota of a critically endangered species: a longitudinal study of kākāpō chick faeces and associated nest litter


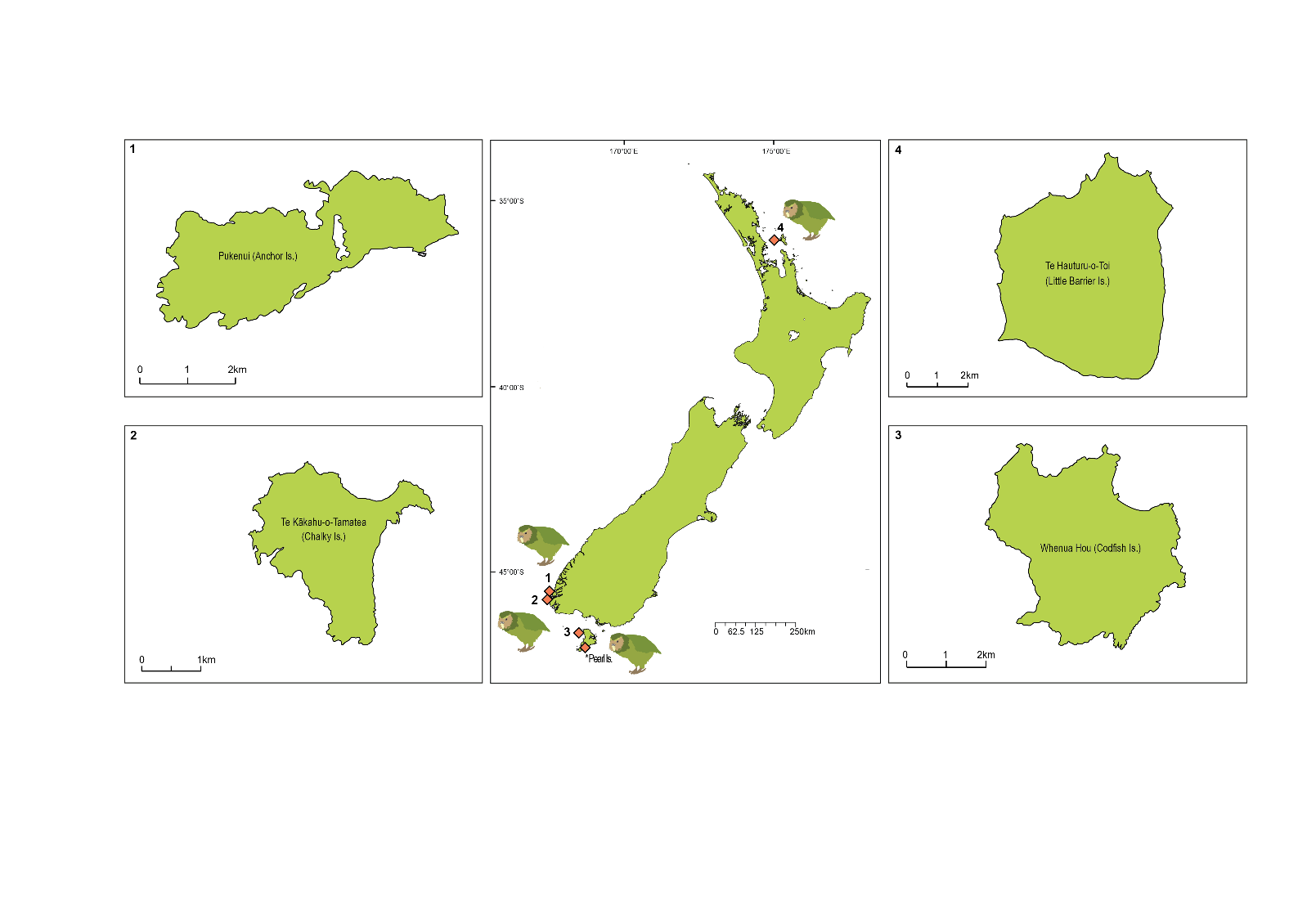


Supplementary Figure 1: Map of Aotearoa New Zealand depicting the offshore islands where kākāpō reside. Faecal and litter samples were collected from Pukenui and Whenua Hou islands as they were the only populations that bred in 2019. Kākāpō were recently re-released on *Pearl Island following the 2019 breeding season.

**Modified DNA extraction protocol**

For each sample, 100 mg of either faecal or litter material was washed once with PBS (to remove RNA*later*) and once with 70% ethanol before suspension in 1 mL of high-salt, CTAB-based extraction buffer (100 mM Tris-HCl (pH 8), 20 mM EDTA, 100 mM sodium phosphate (pH 8), 1.5 M NaCl, 2% CTAB) with 30 mg of polyvinylpolypyrrolidone (PVPP), followed by agitation on a TissueLyser II machine at 30 Hz for 80 s. Lysed samples were incubated at 65°C for 30 min with mixing by inversion every 10 min, followed by a 24:1 chloroform/isoamyl alcohol extraction (500 µL of chloroform/isoamyl alcohol, mixed by vigorous inversion and centrifuged at 13,000 rpm for 10 min). The supernatant (800 µL) was transferred to a new tube containing 0.6 vol isopropanol and incubated at room temperature for 15 min followed by centrifugation at 11,500 rpm at 4°C for 30 min. The isopropanol was subsequently removed and 1 mL of 70% ethanol was added to the tube containing pelleted DNA and left at room temperature for 1 h to absorb excess co-precipitated salts. Samples were then centrifuged for 10 min at 4°C at 13,000 rpm, the supernatant discarded, then the pellet washed again with 1 mL 70% ethanol followed by another 10 min centrifugation (4°C, 13,000 rpm). Pellets were briefly air-dried to remove as much ethanol as possible before adding 20 µL of TE buffer (1 M, pH 8), 30 mg of PVPP and 800 µL of high-salt TE buffer (1.5 M NaCl). Samples were briefly vortexed (1,400 rpm) before the addition of 200 µL of pre-warmed NaCl/CTAB solution (0.7 M and 10%, respectively) then incubated at 65°C with mixing by inversion every 10 min, followed by two rounds of chloroform/isoamyl alcohol extraction. The final supernatant (650 µL) was centrifuged for 2 min at 13,000 rpm to remove remaining PVPP particles and incubated overnight at -20°C with 0.1 vol 3 M sodium acetate (pH 5.2) and 2 vol of 70% ethanol. After two 70% ethanol wash rounds, the final pellet was resuspended in 20 µL of 10 mM Tris-HCl (pH 8).


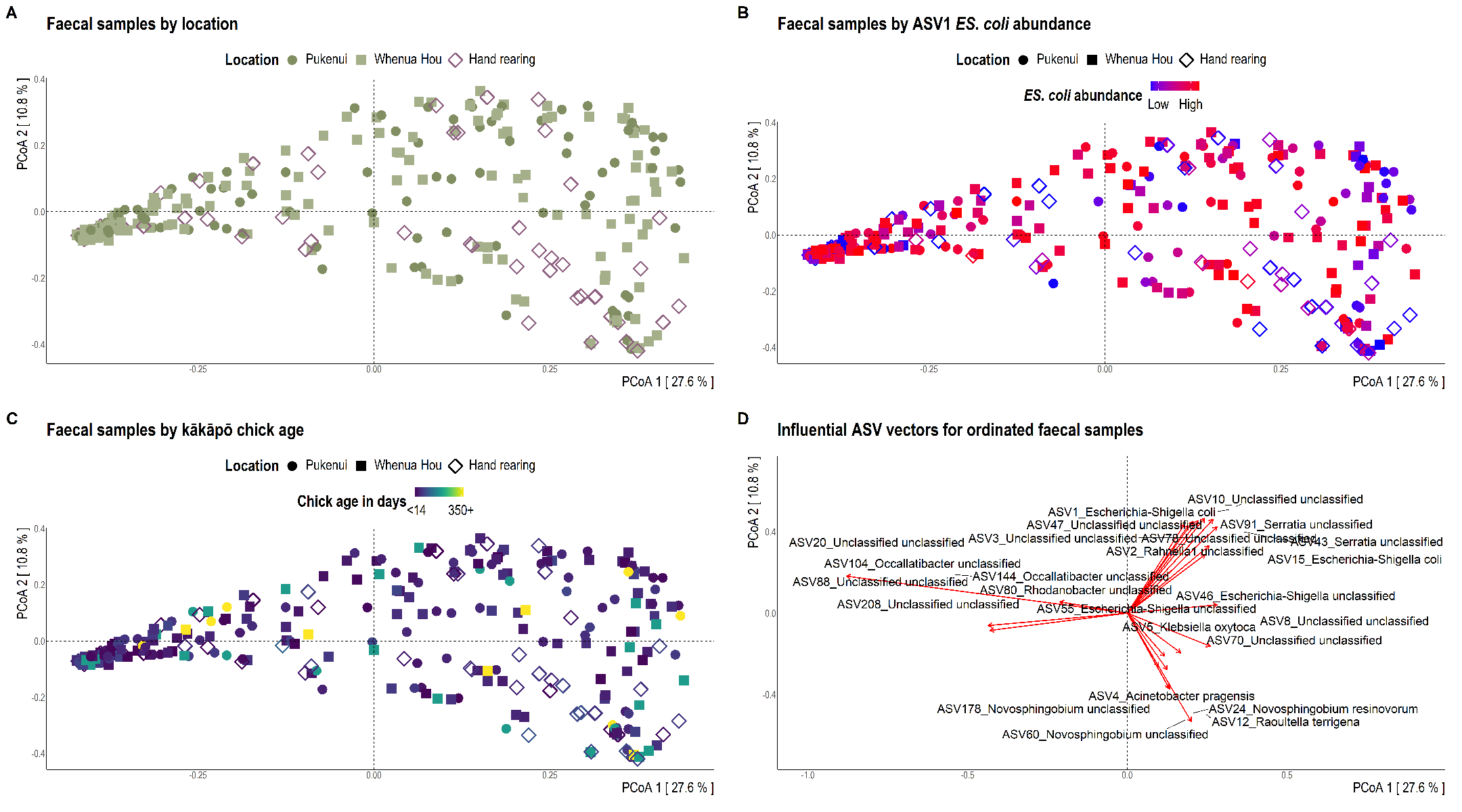


Supplementary Figure 2: gUniFrac dissimilarity distances based on 16S rRNA gene sequences for faecal samples visualised via principal coordinate analysis (PCoA) ordination. Each dot of the PCoA represents the microbiota of a single kākāpō chick faecal sample. Samples are shaped by location and coloured by [A] whether samples were collected while the chick was in the hand rearing facility versus in a nest, [B] relative abundance of Escherichia-Shigella coli, and [C] chick age at sample collection. Panel D depicts the most influential ASV vectors plotted using the vegan::envfit function.


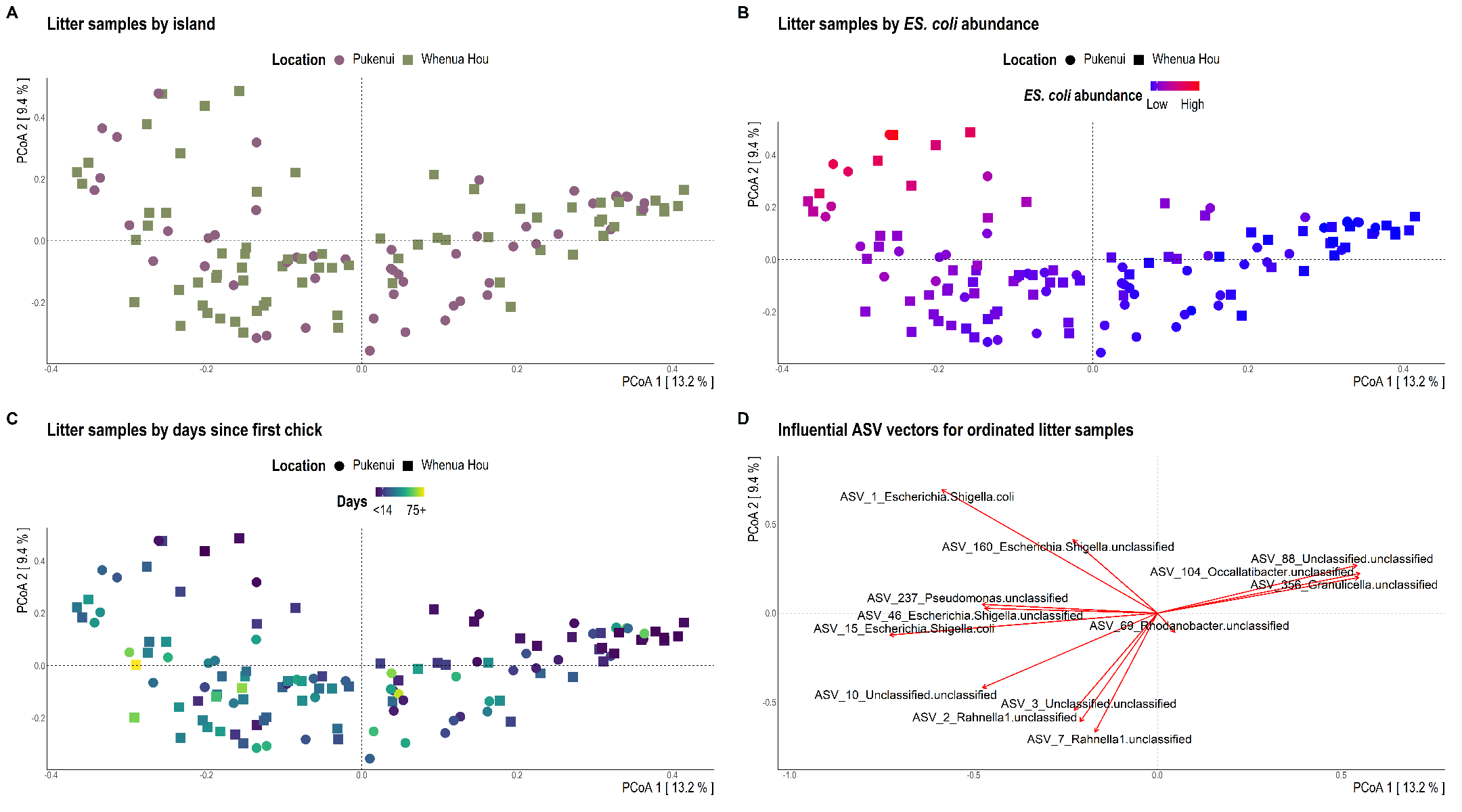


Supplementary Figure 3: gUniFrac dissimilarity distances of 16S rRNA gene sequences for litter samples visualised via principal coordinate analysis ordination (PCoA). Each dot of the PCoA represents the microbiota of a single litter sample. Samples are shaped by island location and coloured by [A] island location, [B] relative abundance of Escherichia-Shigella coli, and [C] number of days since the nest sampled first housed a chick. Panel D depicts the most influential ASV vectors plotted using the vegan::envfit function.


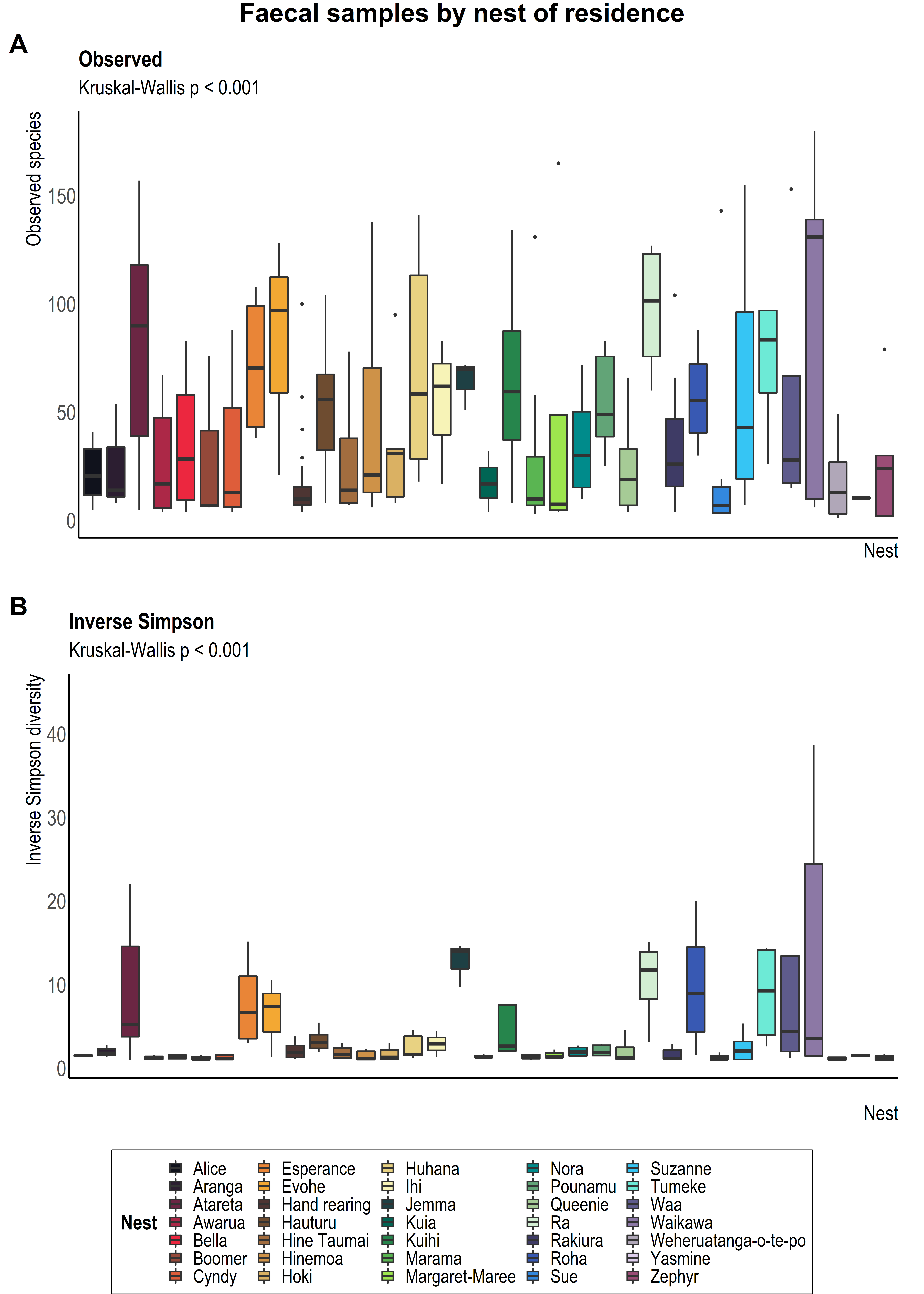


Supplementary Figure 4: [A] Observed richness and [B] Inverse Simpson alpha-diversity indices for kākāpō chick faecal samples grouped by the nest of residence (named after the kākāpō mother tending the nest). Boxes represent the median (within-box horizontal line), 25th (lower hinge) and 75th (upper hinge) percentiles. Whiskers extend to the smallest and largest values within 1.5 times interquartile range above the 25th and 75th percentiles, respectively. Data beyond the end of the whiskers are outlying points and plotted individually for plot A, or excluded for plot B.


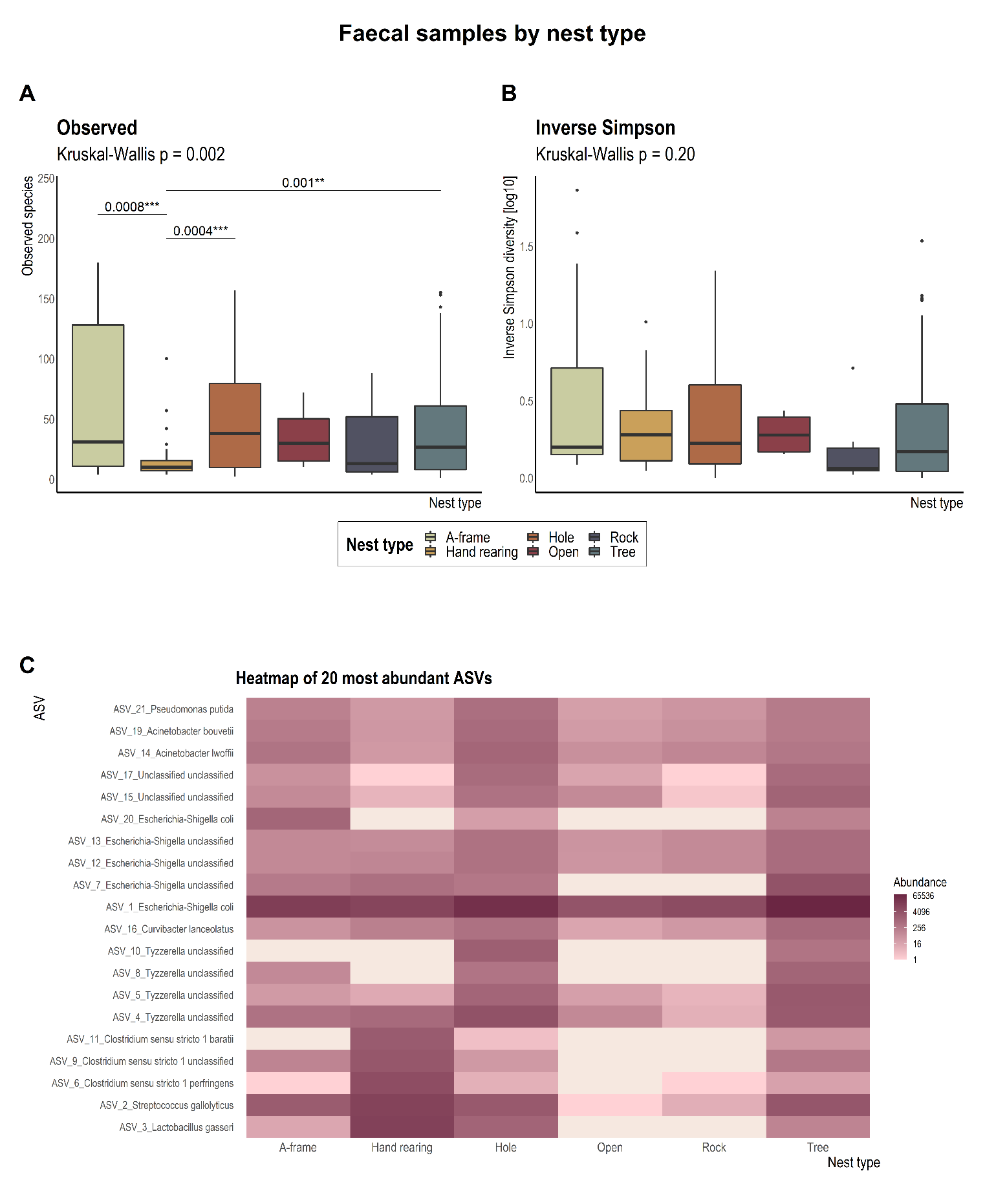


Supplementary Figure 5: [A] Observed and [B] Inverse Simpson alpha-diversity indices for kākāpō chick faecal samples grouped by nest type. Significant Dunn’s test pairwise comparisons with Benjamini-Hochberg adjustment between nest type groups in the box-plot are denoted by asterisks (* = p < 0.05, ** = p < 0.01, *** = p < 0.001). Box-plot details are as described for Supplementary Figure 2. [C] Heatmap highlighting the relative abundance of the 20 most abundant ASVs in faecal samples by nest type. ASV abundances are pooled per group; columns, therefore, have differing total abundances dependent on the number of samples per group (e.g., Rock and Open groups only have 6 and 4 faecal samples, respectively). ASVs are ordered by family-level taxonomy.


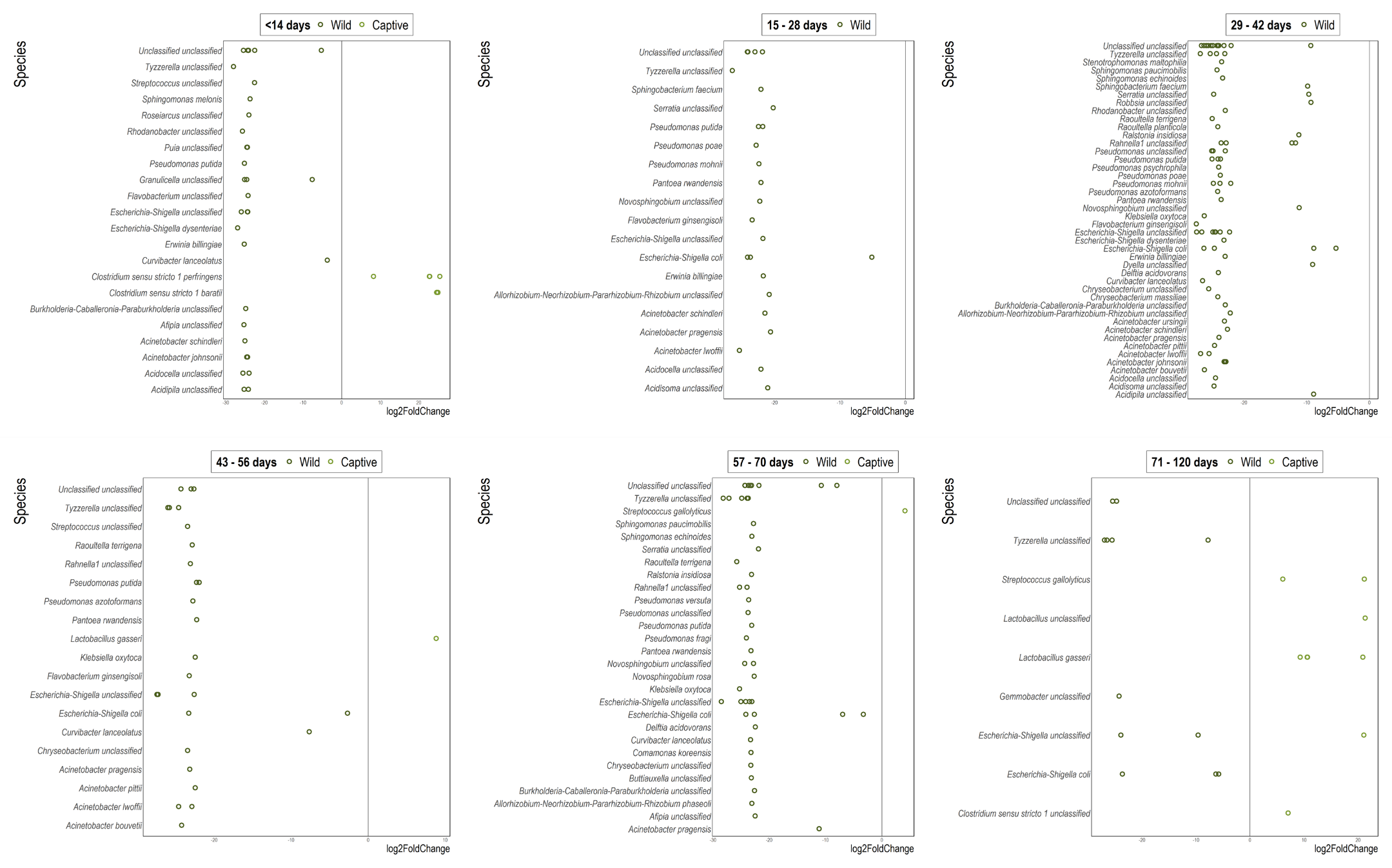


Supplementary Figure 6: Differentially abundant ASVs (p value < 0.01; Wald test with local regression fit and Benjamini–Hochberg p value adjustment) between faecal samples collected from chicks in hand rearing (captive) versus those out in nests (wild) for each age group (200+ days excluded). Each circle represents an individual 16S rRNA gene defined ASV.


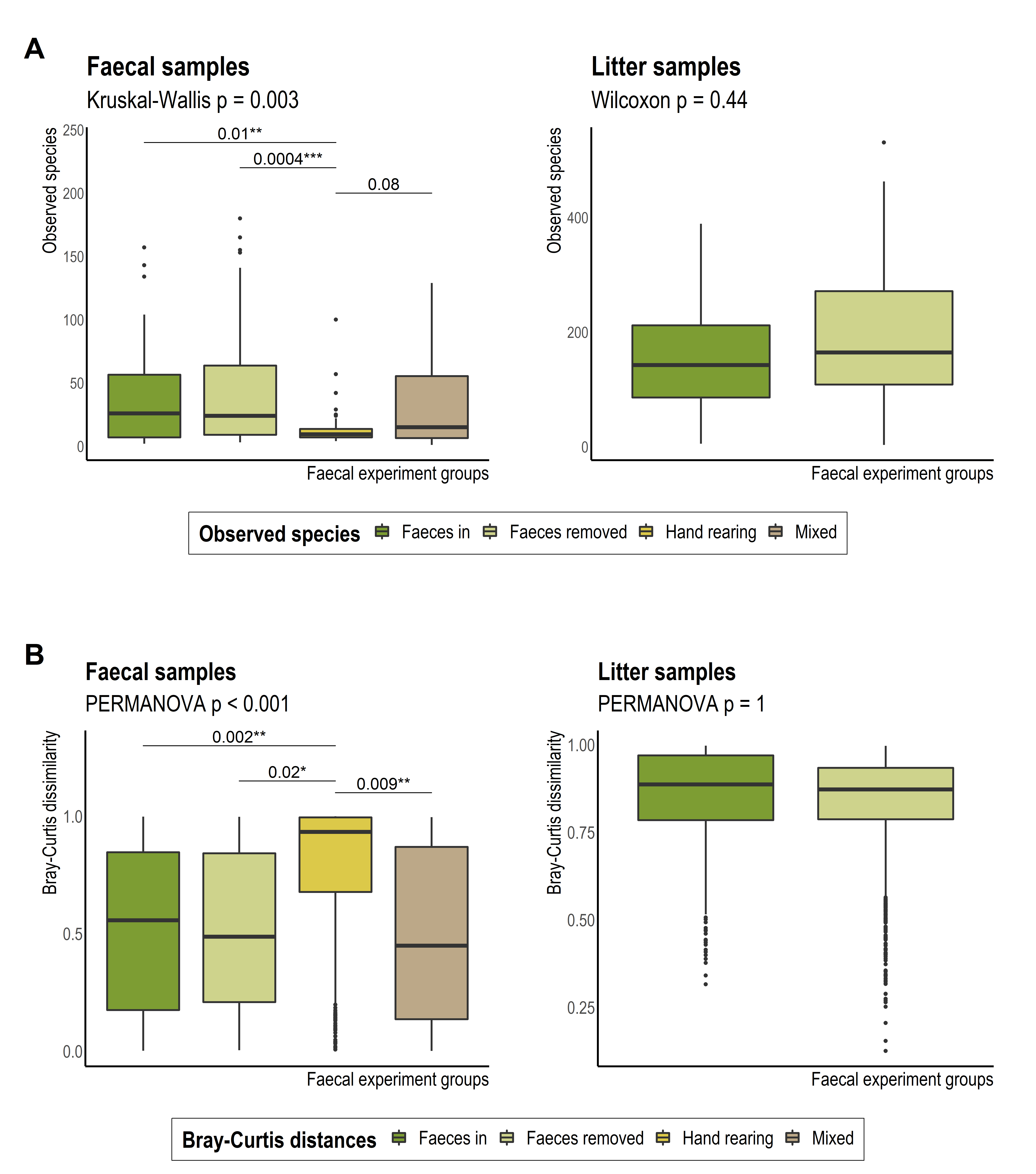


Supplementary Figure 7: [A] Alpha-diversity indices for kākāpō chick faecal samples and litter samples grouped by faecal experiment. Significant Dunn’s test pairwise comparisons with Benjamini-Hochberg adjustment between location groups in the box-plots are shown (p < 0.05 = *, p < 0.01 = **, p < 0.001 = ***). [B] Within-group 16S rRNA gene sequence-based Bray-Curtis dissimilarity distances among samples plotted for faecal and litter samples separately. Significant pairwise PERMANOVA p-values with Benjamini-Hochberg adjustment are shown (p < 0.05 = *, p < 0.01 = **, p < 0.001 = ***). Box-plot details are as described for Supplementary Figure 2.

Supplementary Table 1: Results from the ANOVA- and parametric bootstrapping-based likelihood ratio tests for alpha-diversity estimates. Significant p-values are denoted by asterisks (* = p < 0.05, ** = p < 0.01, *** = p < 0.001). ^ Samples from ‘Unknown’ nest and hand rearing-associated samples were excluded from analyses. # Hand rearing and Sub-adult samples were excluded from analyses. + Hand rearing samples were excluded from analysis. Confidence intervals for Nest type levels are reported overall. For the linear mixed model, the confidence intervals of some Nest types did not cross zero and may thus be significantly associated with Inverse Simpson diversity, though overall the covariate did not meet the alpha threshold.

|  | Generalised linear mixed modelling output | | | | Linear mixed modelling output | | | | |
| --- | --- | --- | --- | --- | --- | --- | --- | --- | --- |
| Covariate | Observed | | | | Inverse Simpson | | | | |
|  | p-value | 2.5% CI | 97.5% CI | ChiSq | p-value | 2.5% CI | 97.5% CI | ChiSq |  |
| ***Faecal samples*** | | | | | | | | |  |
| Faecal removal^ | 0.12 | -0.08 / -0.91 | 1.30 / 0.81 | 4.28 | 0.60 | -0.35 / -0.74 | 0.65 / 0.48 | 1.03 |  |
| Movement^#^ | 0.90 | -2.32 / -1.85 | 1.98 / 1.17 | 0.20 | 0.75 | -1.25 / -0.65 | 0.58 / 0.48 | 0.58 |  |
| Location^+^ | 0.53 | -0.39 | 0.75 | 0.39 | 0.61 | -0.55 | 0.34 | 0.26 |  |
| Aspergillosis | 0.11 | -1.48 / -0.32 | 0.27 / 0.83 | 4.49 | 0.59 | -1.1 / -0.61 | 0.34 / 0.36 | 1.07 |  |
| Age (as continuous variable) | <0.001*** | -0.005 | -0.002 | 47.72 | 0.15 | -0.003 | 0.0005 | 2.10 |  |
| Age as fortnight categories | <0.001*** | <0 | <0 / >0 | 274.06 | 0.09 | <0 | <0 / >0 | 10.90 |  |
| Nest type^#^ | 0.65 | <0 | >0.8 | 2.50 | 0.07 | <0 | <0 / >0 | 8.57 |  |
| Hand rearing | <0.001*** | -0.64 | -0.35 | 46.75 | 0.21 | -0.57 | 0.13 | 1.56 |  |
| ***Litter samples*** | | | | | | | | |  |
| Faecal removal | 0.48 | -0.34 | 0.71 | 0.51 | 0.91 | -0.35 / -1.14 | 0.55 / 1.14 | 0.20 |  |
| Movement | 0.86 | -0.45 | 0.53 | 0.03 | 0.16 | -0.78 | 0.13 | 2.03 |  |
| Island | 0.73 | -0.40 | 0.56 | 0.12 | 0.42 | -0.62 | 0.26 | 0.66 |  |
| Days since first chick (continuous variable) | <0.001*** | -2.89 | -1.07 | 17.28 | 0.002** | -0.021 | -0.005 | 9.74 |  |
| Days as fortnight categories | <0.001*** | <0 | <0 | 22.49 | 0.002** | <0 | <0 | 19.39 |  |
| Aspergillosis | 0.65 | -1.34 / -0.70 | 0.71 / 0.28 | 0.86 | 0.37 | -1.49 / -0.45 | 0.31 / 0.48 | 1.99 |  |
| Nest type | 0.34 | <0 | >0.05 | 4.54 | 0.06 | <0 | >0.5 | 8.93 |  |

*Supplementary Table 2: Results from the betadisper test for homogeneous group dispersion. p-values obtained from the vegan permutest function with 9999 permutations and Benjamini-Hochberg adjusted. Significant p-values are denoted by asterisks (* = p < 0.05, ** = p < 0.01, *** = p < 0.001).*

|  |  | Average distance of samples to group median | | | | | | | |
| --- | --- | --- | --- | --- | --- | --- | --- | --- | --- |
|  |  | Faecal experiment | | | |  |  |  | |
|  | p-value | Faeces in | Faeces removed | Mixed | Hand rearing |  |  |  | |
| Faecal samples | 0.0002*** | 0.34 | 0.34 | 0.32 | 0.57 |  |  |  | |
| Litter samples | 0.79 | 0.60 | 0.60 |  |  |  |  |  | |
|  |  | Chick age / Days since first chick in nest | | | | | | | |
|  |  | <14 days | 15 - 28 days | 29 - 42 days | 43 - 56 days | 57 - 70 days | 71 - 120 days | 200+ days | |
| Faecal samples | 0.0002*** | 0.53 | 0.42 | 0.37 | 0.32 | 0.40 | 0.52 | 0.27 | |
| Litter samples | 0.0006*** | 0.62 | 0.58 | 0.52 | 0.56 | 0.59 | 0.44 |  | |
|  |  | Nest type | | | | | | |  |
|  |  | A-frame | Hole | Open | Rock | Tree | Hand rearing |  | |
| Faecal samples | 0.0002*** | 0.38 | 0.39 | 0.16 | 0.15 | 0.32 | 0.57 |  | |
| Litter samples | 0.003** | 0.59 | 0.59 | 0.47 | 0.44 | 0.60 |  |  | |
|  |  | Movement of chicks | | |  |  |  |  | |
|  |  | No | Yes | Previous |  |  |  |  | |
| Faecal samples | 0.0002*** | 0.52 | 0.36 | 0.20 |  |  |  |  | |
| Litter samples | 0.59 | 0.59 | 0.60 |  |  |  |  |  | |
|  |  | Location | | |  |  |  |  | |
|  |  | Pukenui | Whenua Hou | Hand rearing |  |  |  |  | |
| Faecal samples | 0.0002*** | 0.39 | 0.30 | 0.57 |  |  |  |  | |
| Litter samples | 0.57 | 0.60 | 0.60 |  |  |  |  |  | |
|  |  | Aspergillosis | | |  |  |  |  | |
|  |  | Unaffected | Linked | Infected |  |  |  |  | |
| Faecal samples | 0.04* | 0.44 | 0.31 | 0.36 |  |  |  |  | |
| Litter samples | 0.06 | 0.60 | 0.51 | 0.60 |  |  |  |  | |

*Supplementary Table 3: Statistical output for pairwise comparisons of alpha- and beta-diversity indices against significant covariates identified in Table 3. p-values from both Dunn’s test and pairwise PERMANOVA comparisons are Benjamini-Hochberg adjusted for multiple testing. Significant p-values are denoted by asterisks (* = p < 0.05, ** = p < 0.01, *** = p < 0.001).*

|  | Dunn's adjusted p-values for alpha-diversity | | Pairwise PERMANOVA for Bray-Curtis matrices | | |
| --- | --- | --- | --- | --- | --- |
| Covariate | Observed | Inverse Simpson | p-value | F | R^2^ |
| Faecal samples | | | | | |
| Faecal experiment | | | | | |
| Faeces in vs Faeces removed | 0.19 |  | 1 | 0.96 | 0.005 |
| Faeces in vs Hand rearing | 0.001** |  | 0.002** | 20.98 | 0.16 |
| Faeces in vs Mixed | 0.23 |  | 1 | 0.49 | 0.004 |
| Faeces removed vs Hand rearing | 0.0004*** |  | 0.02** | 31.89 | 0.16 |
| Faeces removed vs Mixed | 0.06 |  | 1 | 0.65 | 0.004 |
| Hand rearing vs Mixed | 0.08 |  | 0.009** | 19.26 | 0.17 |
| Location | | | | | |
| Pukenui vs Whenua Hou | 0.04* |  | 0.54 | 4.71 | 0.02 |
| Pukenui vs Hand rearing | 0.0003*** |  | 0.0001*** | 23.69 | 0.15 |
| Whenua Hou vs Hand rearing | 0.007** |  | 0.0001*** | 41.76 | 0.18 |
| Age | | | | | |
| <14 days vs 15 - 28 days | 0.12 |  | 0.21 | 2.39 | 0.03 |
| <14 days vs 29 - 42 days | 0.04* |  | 0.003** | 5.25 | 0.05 |
| <14 days vs 43 - 56 days | 0.15 |  | <0.001*** | 5.44 | 0.06 |
| <14 days vs 57 - 70 days | 0.47 |  | 0.009** | 3.20 | 0.04 |
| <14 days vs 71 - 120 days | 0.008** |  | 0.53 | 3.29 | 0.04 |
| <14 days vs 200+ days | 0.002** |  | <0.001*** | 8.45 | 0.08 |
| 15 - 28 days vs 29 - 42 days | 0.34 |  | 0.15 | 0.87 | 0.009 |
| 15 - 28 days vs 43 - 56 days | 0.02* |  | 0.36 | 1.93 | 0.02 |
| 15 - 28 days vs 57 - 70 days | 0.15 |  | 0.08 | 1.39 | 0.02 |
| 15 - 28 days vs 71 - 120 days | 0.0006*** |  | 1 | 3.86 | 0.06 |
| 15 - 28 days vs 200+ days | <0.0001*** |  | 0.009** | 4.19 | 0.06 |
| 29 - 42 days vs 43 - 56 days | 0.003** |  | 0.05 | 1.22 | 0.01 |
| 29 - 42 days vs 57 - 70 days | 0.07 |  | 0.65 | 1.92 | 0.02 |
| 29 - 42 days vs 71 - 120 days | 0.0001*** |  | 0.41 | 5.37 | 0.07 |
| 29 - 42 days vs 200+ days | <0.0001*** |  | <0.001*** | 4.53 | 0.05 |
| 43 - 56 days vs 57 - 70 days | 0.16 |  | 0.008** | 1.38 | 0.02 |
| 43 - 56 days vs 71 - 120 days | 0.07 |  | 0.10 | 4.29 | 0.07 |
| 43 - 56 days vs 200+ days | 0.04* |  | 0.03* | 2.45 | 0.03 |
| 57 - 70 days vs 71 - 120 days | 0.02* |  | 0.006** | 3.06 | 0.06 |
| 57 - 70 days vs 200+ days | 0.004** |  | 0.002** | 4.05 | 0.05 |
| 71 - 120 days vs 200+ days | 0.49 |  | 0.31 | 8.94 | 0.12 |
| Nest type | | | | | |
| Hand rearing vs A-frame | 0.0008*** | 0.31 | 0.004** | 9.88 | 0.13 |
| Hand rearing vs Hole | 0.0004*** | 0.45 | 0.04* | 17.41 | 0.14 |
| Hand rearing vs Open | 0.25 | 0.49 | 1 | 3.91 | 0.08 |
| Hand rearing vs Rock | 0.33 | 0.22 | 0.49 | 5.48 | 0.10 |
| Hand rearing vs Tree | 0.001** | 0.20 | 0.0002*** | 32.44 | 0.18 |
| A-frame vs Hole | 0.40 | 0.36 | 1 | 0.90 | 0.01 |
| A-frame vs Open | 0.45 | 0.47 | 1 | 0.83 | 0.03 |
| A-frame vs Rock | 0.29 | 0.22 | 1 | 1.05 | 0.04 |
| A-frame vs Tree | 0.32 | 0.27 | 0.02* | 1.84 | 0.02 |
| Hole vs Open | 0.50 | 0.50 | 1 | 0.97 | 0.02 |
| Hole vs Rock | 0.28 | 0.20 | 1 | 1.11 | 0.02 |
| Hole vs Tree | 0.35 | 0.16 | 0.35 | 2.91 | 0.02 |
| Open vs Rock | 0.34 | 0.30 | 1 | 1.30 | 0.14 |
| Open vs Tree | 0.45 | 0.37 | 1 | 0.94 | 0.009 |
| Rock vs Tree | 0.35 | 0.34 | 0.51 | 0.79 | 0.007 |
| Litter samples | | | | | |
| Days since first chick in nest | | | | | |
| <14 days vs 15 - 28 days | 0.05* | 0.05* | 0.0004*** | 2.43 | 0.04 |
| <14 days vs 29 - 42 days | 0.007** | 0.05* | 0.0008*** | 4.23 | 0.07 |
| <14 days vs 43 - 56 days | 0.004** | 0.08 | 0.0001*** | 3.71 | 0.06 |
| <14 days vs 57 - 70 days | 0.21 | 0.51 | 0.04* | 1.39 | 0.03 |
| <14 days vs 71 - 90 days | 0.11 | 0.16 | 0.08 | 3.14 | 0.07 |
| 15 - 28 days vs 29 - 42 days | 0.35 | 0.52 | 0.03* | 1.39 | 0.03 |
| 15 - 28 days vs 43 - 56 days | 0.21 | 0.52 | 0.0005*** | 1.67 | 0.03 |
| 15 - 28 days vs 57 - 70 days | 0.50 | 0.14 | 0.70 | 1.15 | 0.03 |
| 15 - 28 days vs 71 - 90 days | 0.42 | 0.55 | 0.08 | 2.33 | 0.06 |
| 29 - 42 days vs 43 - 56 days | 0.44 | 0.57 | 0.25 | 1.09 | 0.03 |
| 29 - 42 days vs 57 - 70 days | 0.43 | 0.13 | 0.29 | 1.61 | 0.05 |
| 29 - 42 days vs 71 - 90 days | 0.52 | 0.50 | 0.11 | 1.61 | 0.06 |
| 43 - 56 days vs 57 - 70 days | 0.33 | 0.15 | 0.21 | 1.12 | 0.04 |
| 43 - 56 days vs 71 - 90 days | 0.45 | 0.55 | 0.75 | 1.15 | 0.04 |
| 57 - 70 days vs 71 - 90 days | 0.43 | 0.18 | 0.33 | 1.62 | 0.12 |

Supplementary Table 4: Estimates obtained from linear mixed models fitting age and location covariates against the log-transformed relative abundances of key bacterial genera present in faecal samples. p-values (Benjamini-Hochberg adjusted) represent likelihood ratio tests comparing a null model with the target model for each covariate and genus with ANOVA.

|  | *Escherichia-Shigella* | | | | | | | | | |
| --- | --- | --- | --- | --- | --- | --- | --- | --- | --- | --- |
|  | Location p = 1e^-10^*** chi = 55.82 | | | |  | |  | |  | |
|  | Hand rearing (Intercept) | Pukenui | Whenua Hou |  | |  | |  | |  |
| Estimate | 4.64 | 1.92 | 2.28 |  | |  | |  | |  |
| Standard Error | 0.31 | 0.32 | 0.29 |  | |  | |  | |  |
| t value | 15.02 | 6.10 | 7.87 |  | |  | |  | |  |
|  | Age p = 6.7e^-05^*** chi = 30.09 | | | | | | | | | |
|  | <14 days (Intercept) | 15 - 28 days | 29 - 42 days | 43 - 56 days | | 57 – 70 days | | 71 - 120 days | | 200+ days |
| Estimate | 5.75 | 0.79 | 0.98 | 1.35 | | 1.21 | | 0.29 | | 0.86 |
| Standard Error | 0.26 | 0.29 | 0.27 | 0.29 | | 0.31 | | 0.37 | | 0.86 |
| t value | 22.00 | 2.72 | 3.62 | 4.75 | | 3.90 | | 0.68 | | 1.00 |
|  | *Streptococcus* | | | | | | | | | |
|  | Location p = 5.7e^-04^*** chi = 15.65 | | |  | |  | | | |  |
|  | Hand rearing (Intercept) | Pukenui | Whenua Hou |  | |  | |  | |  |
| Estimate | 3.27 | -1.46 | -1.65 |  | |  | |  | |  |
| Standard Error | 0.37 | 0.42 | 0.39 |  | |  | |  | |  |
| t value | 8.76 | -3.47 | -4.20 |  | |  | |  | |  |
|  | Age p = 0.19 chi = 8.80 | | | | | | | | | |
|  | <14 days (Intercept) | 15 - 28 days | 29 - 42 days | 43 - 56 days | | 57 – 70 days | | 71 - 120 days | | 200+ days |
| Estimate | 2.18 | 0.20 | -0.70 | -0.46 | | 0.37 | | -0.16 | | -1.03 |
| Standard Error | 0.33 | 0.45 | 0.42 | 0.44 | | 0.48 | | 0.56 | | 0.87 |
| t value | 6.53 | 0.44 | -1.68 | -1.05 | | 0.76 | | -0.28 | | -1.19 |
|  | *Lactobacillus* | | | | | | | | | |
|  | Location p = 1e^-15^*** chi = 72.64 | | |  | |  | | | |  |
|  | Hand rearing (Intercept) | Pukenui | Whenua Hou |  | |  | |  | |  |
| Estimate | 3.89 | -3.37 | -3.45 |  | |  | |  | |  |
| Standard Error | 0.36 | 0.33 | 0.31 |  | |  | |  | |  |
| t value | 10.87 | -10.13 | -11.19 |  | |  | |  | |  |
|  | Age p = 4e^-08^*** chi = 46.65 | | | | | | | | | |
|  | <14 days (Intercept) | 15 - 28 days | 29 - 42 days | 43 - 56 days | | 57 – 70 days | | 71 - 120 days | | 200+ days |
| Estimate | -0.60 | 1.35 | 1.59 | 1.58 | | 1.71 | | 2.78 | | 0.18 |
| Standard Error | 0.29 | 0.33 | 0.31 | 0.33 | | 0.35 | | 0.41 | | 0.97 |
| t value | -2.04 | 4.11 | 5.16 | 4.87 | | 4.83 | | 6.71 | 0.19 | |
|  | *Tyzzerella* | | | | | | | | | |
|  | Location p = 1.3e^-09^*** chi = 42.90 | | |  | |  | | |  | |
|  | Hand rearing (Intercept) | Pukenui | Whenua Hou |  | |  | |  |  | |
| Estimate | 0.52 | 2.96 | 1.95 |  | |  | |  |  | |
| Standard Error | 0.45 | 0.41 | 0.38 |  | |  | |  |  | |
| t value | 1.16 | 7.28 | 5.16 |  | |  | |  |  | |
|  | Age p = 0.03* chi = 13.73 | | | | | | | | | |
|  | <14 days (Intercept) | 15 - 28 days | 29 - 42 days | 43 - 56 days | | 57 – 70 days | | 71 - 120 days | 200+ days | |
| Estimate | 2.27 | -0.10 | -0.13 | 0.30 | | 0.46 | | -0.10 | 3.06 | |
| Standard Error | 0.34 | 0.43 | 0.40 | 0.41 | | 0.46 | | 0.54 | 0.93 | |
| t value | 6.77 | -0.24 | -0.32 | 0.72 | | 1.01 | | -0.19 | 3.28 | |
| *Clostridium sensu stricto 1* | | | | | | | | | | |
|  | Location p = 1e^-15^*** chi = 104.99 | | |  | |  | |  |  | |
|  | Hand rearing (Intercept) | Pukenui | Whenua Hou |  | |  | |  |  | |
| Estimate | 3.27 | -3.41 | -3.13 |  | |  | |  |  | |
| Standard Error | 0.32 | 0.33 | 0.31 |  | |  | |  |  | |
| t value | 10.32 | -10.28 | -10.28 |  | |  | |  |  | |
|  | Age p =0.001** chi = 22.06 | | | | | | | | | |
|  | <14 days (Intercept) | 15 - 28 days | 29 - 42 days | 43 - 56 days | | 57 – 70 days | | 71 - 120 days | 200+ days | |
| Estimate | 1.03 | -1.16 | -0.95 | -1.13 | | 0.97 | | -1.52 | -0.17 | |
| Standard Error | 0.27 | 0.33 | 0.31 | 0.32 | | 0.36 | | 0.42 | 0.77 | |
| t value | 3.76 | -3.49 | -3.08 | -3.52 | | -2.71 | | -3.63 | -0.22 | |
